# Tetrandrine-driven autophagy suppresses SARS-CoV-2 replication by modulating cholesterol and IGF signaling pathways

**DOI:** 10.1038/s41420-025-02926-7

**Published:** 2026-01-06

**Authors:** Lais de O. Marchioro, Sofia De Stefanis, Beatriz G. Araújo, Davide Mariotti, Ingrid K. M. Watanabe, Michael Stumpe, Giulia Matusali, Fabrizio Maggi, Soraya S. Smaili, Jörn Dengjel, Gustavo J. S. Pereira, Manuela Antonioli

**Affiliations:** 1 Department of Pharmacology, Escola Paulista de Medicina, Universidade Federal de São Paulo, São Paulo, SP Brazil; 2 Department of Epidemiology, Preclinical Research and Advanced Diagnostics, National Institute for Infectious Diseases “Lazzaro Spallanzani”- IRCCS 00149, Rome, Italy; 3 PhD Program in Cellular and Molecular Biology, Department of Biology, University of Rome “Tor Vergata”, 00133 Rome, Italy; 4 Laboratory of Virology and Laboratories of Biosafety, National Institute for Infectious Diseases “Lazzaro Spallanzani”-IRCCS, 00149 Rome, Italy; 5 Nephrology Division, Department of Medicine, Escola Paulista de Medicina, Universidade Federal de São Paulo, São Paulo, SP Brazil; 6 Department of Biology, University of Fribourg, 1700 Fribourg, Switzerland; 7 Department of Cell and Developmental Biology, University College London, London, UK; 8 Department of Biology, University of Rome “Tor Vergata”, 00133 Rome, Italy

**Keywords:** Autophagy, Target identification, Antiviral agents

## Abstract

SARS-CoV-2 exploits multiple host cellular processes, including autophagy, a critical intracellular degradation pathway, to facilitate viral replication and evade immune detection. Tetrandrine, a natural bis-benzylisoquinoline alkaloid derived from *Stephania tetrandra*, has been reported to modulate autophagy and exhibits potential antiviral properties. In this study, we investigated the effects of Tetrandrine on SARS-CoV-2 infection in human lung epithelial cells (Calu-3), with a particular focus on autophagy-related mechanisms. Our results demonstrate that Tetrandrine modulates autophagic activity in a dose-dependent manner and significantly reduces SARS-CoV-2 replication, particularly when administered prior to infection. Notably, its antiviral effect is retained in autophagy-deficient cells, indicating the involvement of autophagy-independent mechanisms. Proteomic analysis of Calu-3 cells infected with the Omicron BA.5 variant revealed that Tetrandrine regulates several host pathways implicated in viral replication, including autophagy, cholesterol metabolism, and insulin-like growth factor signaling. These findings suggest that Tetrandrine exerts multifaceted antiviral effects by targeting both autophagy-dependent and -independent cellular pathways. Collectively, our data supports the potential of Tetrandrine as a therapeutic candidate against COVID-19 and warns further evaluation in preclinical and clinical models. Data are available via ProteomeXchange with identifier PXD064448.

## Introduction

The COVID-19 pandemic, caused by the severe acute respiratory syndrome coronavirus 2 (SARS-CoV-2), has profoundly impacted global health, economies, and healthcare systems. Since its emergence in December 2019, SARS-CoV-2 has rapidly spread worldwide, leading the World Health Organization (WHO) to declare it a pandemic in March 2020 [1, 2]. Despite the development of vaccines and antiviral therapies, SARS-CoV-2 continues to pose a significant threat, particularly to individuals with comorbidities and immunosuppressive conditions. The virus is highly adaptable, with frequent mutations giving rise to variants of concern (VOCs) that can evade immune responses and, in some cases, exhibit increased transmissibility or resistance to existing treatments [3, 4]. Consequently, there is an ongoing need to identify novel therapeutic strategies that can effectively inhibit viral replication and mitigate disease progression. A defining feature of SARS-CoV-2 is its capacity to exploit host cellular processes to promote replication and evade immune responses. Among these, autophagy, an evolutionarily conserved intracellular degradation pathway, plays a pivotal role in host-virus interactions [5]. SARS-CoV-2 manipulates autophagy to facilitate its replication by inducing autophagosome formation while preventing their fusion with lysosomes [6–8]. This blockade, mediated by viral proteins such as ORF3a and ORF7a, inhibits autophagic flux and impairs the degradative steps required for the clearance of viral components, thereby creating a cellular environment that supports viral persistence and immune evasion [9–11]. Owing to this dual function of autophagy in SARS-CoV-2 infection, serving both as a host defense mechanism and as a pathway subverted by the virus, it has been characterized as a double-edged sword in COVID-19 pathogenesis. This complexity poses a major challenge for the development of autophagy-targeting therapeutics, as both autophagy inducers and inhibitors have been proposed to interfere with viral replication [12–15]. A deeper understanding of the mechanisms governing autophagy modulation during SARS-CoV-2 infection is therefore critical for the design of effective antiviral strategies.

One promising compound that has garnered attention for its autophagy-modulating and antiviral properties is Tetrandrine, a bis-benzylisoquinoline alkaloid derived from *Stephania tetrandra*. Traditionally used in Chinese medicine for its anti-inflammatory and immunomodulatory effects, Tetrandrine has been extensively studied for its pharmacological activities, including its ability to regulate calcium signaling, apoptosis and autophagy [16]. It has been previously demonstrated that Tetrandrine exhibits antiviral effects against various pathogens, including herpes simplex virus (HSV-1), human immunodeficiency virus (HIV), Ebola virus, and MERS-CoV [17–20]. More recently, Tetrandrine has been described as inhibiting SARS-CoV-2 replication in vitro, making it a compelling candidate for further investigation [21].

Mechanistically, Tetrandrine modulates autophagy in a dose-dependent manner. At lower concentrations, it has been shown to induce autophagy, promoting the clearance of damaged cellular components. However, at higher concentrations, it inhibits autophagic flux, preventing the fusion of autophagosomes with lysosomes by its ability to antagonize calcium channels like Two-Pore Channel type 2 (TPC2), which may disrupt viral replication processes [22–24]. This dual effect suggests that Tetrandrine could either enhance the antiviral properties of autophagy or impair the virus’s ability to exploit autophagic pathways, depending on the treatment conditions. However, the precise molecular mechanisms by which Tetrandrine exerts its antiviral effects against SARS-CoV-2 remain incompletely understood. Furthermore, its anti-inflammatory properties may be beneficial in mitigating the excessive immune responses and cytokine storm associated with severe COVID-19 cases.

Despite these promising findings, several questions remain regarding the exact molecular targets of Tetrandrine in the context of SARS-CoV-2 infection. Moreover, while in vitro studies suggest potent antiviral activity, further preclinical and clinical evaluations are required to determine its efficacy and safety in COVID-19 patients [25]. Understanding how Tetrandrine modulates autophagy in SARS-CoV-2-infected cells and whether its antiviral effects are primarily autophagy-dependent or involve additional pathways are essential for its potential therapeutic application.

This study aims to investigate the role of Tetrandrine as a therapeutic modulator of autophagy in SARS-CoV-2 infection, focusing on elucidating its molecular mechanisms of action. Specifically, we assess whether Tetrandrine’s antiviral effects are mediated through autophagy regulation or alternative cellular pathways. By combining virological assays, confocal microscopy, and proteomic analyses, we seek to provide a deeper understanding of how Tetrandrine interferes with SARS-CoV-2 replication.

## Results

### Tetrandrine modulates autophagy in Calu-3 cells and reduces SARS-CoV-2 infection

It has been previously shown that SARS-CoV-2-infected cells initially activate autophagy as a survival mechanism to immediately constrain the infection; however, similarly to other viruses, SARS-CoV-2 suddenly inhibits autophagy during infection [26]. Before focusing on Calu-3 cells, we performed an initial screening experiment in Vero-E6 cells, which are widely used as a permissive model for SARS-CoV-2 infection. The aim was to evaluate whether different modulators of two-pore calcium channels (TPCs) could affect viral infectivity. TPCs are endolysosomal Ca²⁺ channels involved in vesicular trafficking, and their activity has been implicated in the entry and propagation of several viruses, including Ebola virus and other coronaviruses. For this reason, we tested NAADP-AM (agonist), Ned-19 (antagonist), and Tetrandrine, which also acts as a broad TPC blocker in addition to modulating autophagy. As shown in Supplementary Fig. 1A and B, only Tetrandrine significantly reduced viral replication in both pre- and post-treatment conditions, while NAADP-AM and Ned-19 had no consistent effect. These results suggested that TPC signaling is not a critical bottleneck for SARS-CoV-2 propagation in this system and guided us to concentrate subsequent analyses on Tetrandrine in Calu-3 cells. Before examining the antiviral effects of Tetrandrine, we tested its toxicity on Calu-3 cells to ensure reduced viral replication was not due to impaired cell growth or survival. To this aim, cell proliferation (MTT assay) and viability (Trypan Blue exclusion) were used following 24 h of treatment with 5 µM and 10 µM Tetrandrine. As shown, no significant changes were detected in either parameter, indicating that the compound does not compromise Calu-3 viability at the concentrations used (Supplementary Fig. 1C, D).

**Fig. 1:**
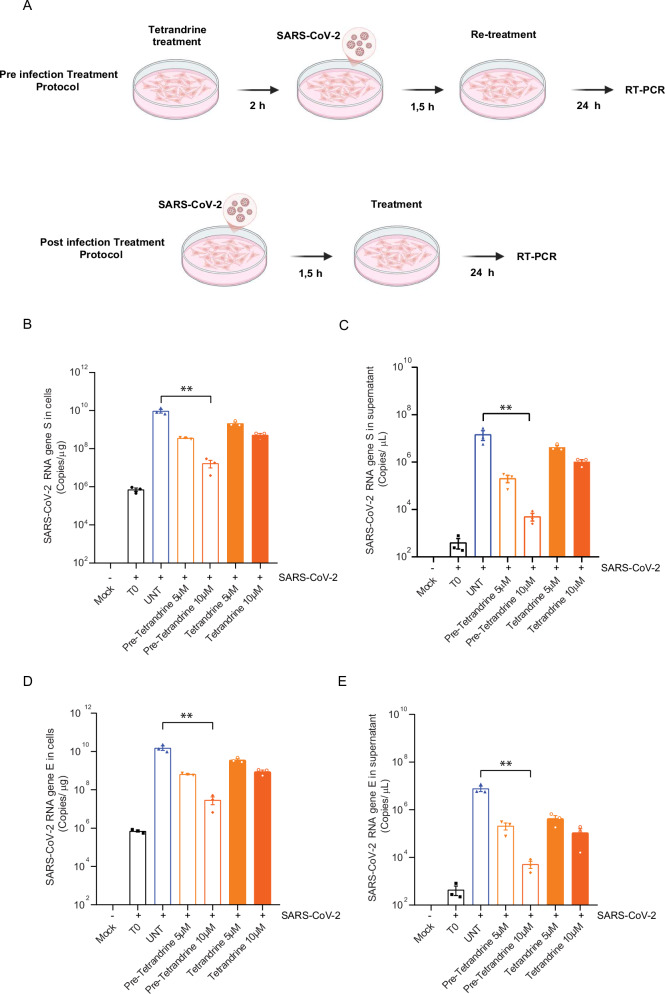
Analysis of Omicron BA.5 SARS-CoV-2 replication in Calu-3 cells treated with Tetrandrine. Quantitative real-time analysis of SARS-CoV-2 BA.5 replication in Calu-3 cells infected and/or pre-treated for two hours with (5 and 10 µM) Tetrandrine. **A** SARS-CoV-2 infection and treatment protocol design; **B** Number of copies/µL of the Spike gene in the intracellular medium after 24 h of treatment; **C** Number of copies/µL of the S gene in the extracellular medium after 24 h of treatment; **D** Number of copies of the E gene per µg of RNA extracted from cells after 24 h with prior treatment; **E** Number of copies of the E gene per µg of RNA extracted from the supernatant after 24 h with prior treatment. All results are expressed as mean ± SEM and analyzed by one-way ANOVA followed by Tukey’s post hoc test. *p < 0.02; ***p < 0.0004; and ****p < 0.0001 compared to the UNT group.

Next, to determine whether Tetrandrine modulates autophagy in lung epithelial cells, we analyzed LC3 lipidation in the presence or absence of the lysosomal inhibitor Bafilomycin A1. Since LC3-I and LC3-II quantification alone can be misleading as readouts of autophagy, the use of Bafilomycin A1 was essential to assess autophagic flux, in accordance with current guidelines [27]. Increased LC3-II accumulation in Bafilomycin A1-treated cells is consistent with autophagy induction; by contrast, no further LC3-II accumulation upon Bafilomycin A1 co-treatment indicates an inhibition of the autophagic flux. Therefore, as shown in Supplementary Fig. 1E, 5 µm Tetrandrine enhances autophagy, whereas 10 µm Tetrandrine inhibits autophagic flux since LC3II accumulation does not increase following lysosomal inhibition. Overall, these results indicate that Tetrandrine differentially regulates autophagy in Calu-3 cells in a dose-dependent manner, thus promoting autophagy at lower concentrations and inhibiting autophagic flux at higher ones, without impacting cell viability. Subsequently, to determine whether Tetrandrine primarily impacts early or late stages of the SARS-CoV-2 cycle, we infected Calu-3 cells with the Omicron BA.5 variant at a multiplicity of infection (MOI) of 0.2 and treated them with Tetrandrine either 2 h before (pre-treatment) and/ or immediately after infection (post-treatment). Then, viral RNA levels were quantified by qPCR in both cells and supernatants 24 h post-infection, evaluating both viral genes S and E (Fig. 1A). Not infected cells were used as a negative control. As shown, both 5 µM and 10 µM Tetrandrine significantly reduced intracellular SARS-CoV-2 RNA, with the strongest effect observed at 10 µM (Figs. 1B and 1D). Moreover, the inhibitory effect on viral infection was even more evident when cells were pre-treated with Tetrandrine compared to post-treated ones. Of note, Fig. 1C and E report the quantity of viral particles released. Similarly, SARS-CoV-2 gene S and E decreases when Tetrandrine is added. However, the post-treatment with Tetrandrine 5 µM did not show any significant difference from the control. Accordingly, the quantity of SARS-CoV-2, analyzed by evaluating gene E by qPCR, showed a similar result to gene S in both cells and supernatants (Fig. 1D, E). Altogether, our results suggest Tetrandrine reduces SARS-CoV-2 infection with higher efficacy when administered before the infection. Therefore, we have performed our subsequent analyses by pre-treating Calu-3 cells with Tetrandrine 2 h before SARS-CoV-2 infection.

### Tetrandrine inhibits Calu-3 infection by blocking SARS-CoV-2 replication

**Fig. 2:**
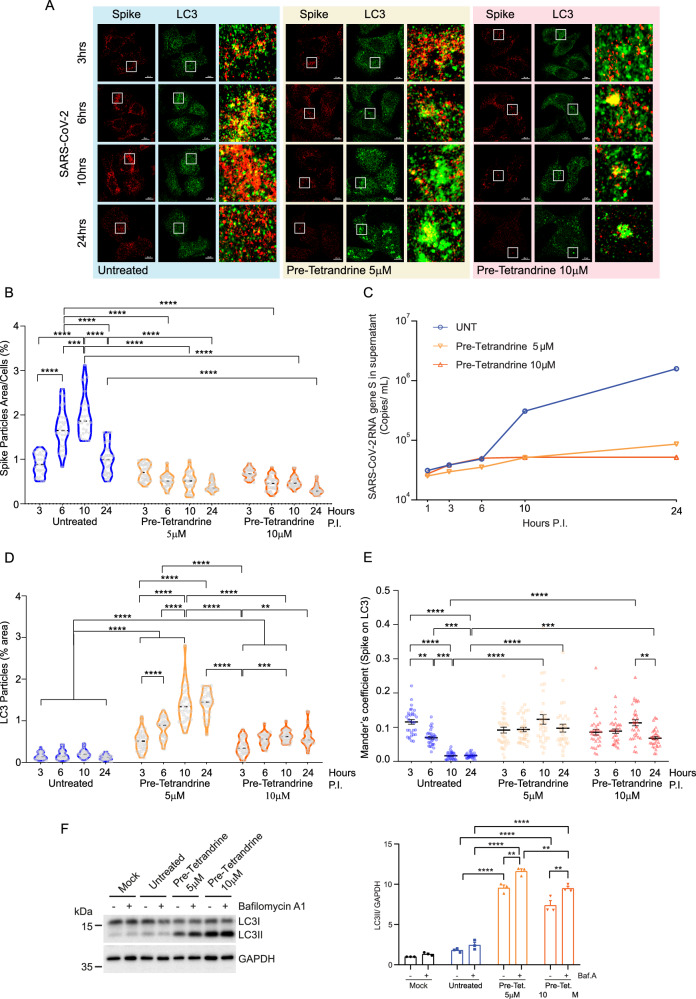
Viral replication and autophagy dynamics in Calu-3 cells infected with the SARS-CoV-2 BA.5 variant. **A** Representative immunofluorescence images of cellular LC3 (green) and viral Spike (red) in cells pre-treated with Tetrandrine at 5 or 10 µM at different time points post-infection (P.I.) with SARS-CoV-2 BA.5. Scale: 10 µm; **B** Quantification of Spike-positive area per cell expressed as a percentage of the total cellular area; **C** Quantitative real-time PCR of the S gene in the supernatant at different time points P.I.; **D** Quantification of LC3-positive area per cell expressed as a percentage of the total cellular area; **E** Mander’s coefficient for Spike localization in LC3; **F** Evaluation of autophagic flux 24 h P.I. in cells pre-treated with Tetrandrine at 5 and 10 µM. Bafilomycin A1 was added 2 h before the lysis, and LC3 and HSP90 were analyzed by Western blotting using anti-LC3 (14 kDa) and anti-HSP90 (90 kDa) antibodies. The optical density representing the relative expression of each protein was normalized using internal control for the anti-HSP90 (90 kDa) antibody, as shown in the representations. All results are expressed as mean ± SEM and analyzed by one-way ANOVA followed by Tukey’s post hoc test. **p < 0.003; ***p < 0.0004; and ****p < 0.0001 compared to the UNT group.

To better define how Tetrandrine affects viral cycle, we analyzed viral protein expression at different time points post-infection by immunofluorescence. This approach was designed to evaluate whether Tetrandrine pre-treated cells are still permissive for viral entry and to evaluate both viral replication and release. To achieve this aim, Calu-3 cells were pre-treated with Tetrandrine for 2 h, then infected with the SARS-CoV-2 BA.5 variant in the presence or absence of Tetrandrine at different time points (3, 6, 10, and 24 h). Following the treatment, cells were fixed in 4% PFA for subsequent immunofluorescence analysis, while the supernatant was collected to monitor viral release by qPCR. To check the contribution of autophagy both on viral life-cycle and on Tetrandrine action, we marked cellular autophagic vesicles (*i.e*. LC3B staining) and viral particles (*i.e*. Spike, S viral protein staining), and subsequently analyze their co-localization by confocal microscopy. As shown in Fig. 2A and B, we observed a similar amount of cytosolic viral Spike (S) protein 3 h post-infection (P.I.) when comparing Tetrandrine treated and untreated cells, indicating the viral uptake is not inhibited by the treatment. Differently, during viral replication at prolonged time points, Tetrandrine treated cells differ significantly from untreated ones. In particular, untreated Calu-3 cells showed high levels of cytosolic viral Spike (S) 6 and 10 h P.I. with a subsequent decrease at 24 h P.I., which corresponds to the viral particles release, as observed by qPCR in the supernatant (Fig. 2C, blue line). In contrast, Tetrandrine-treated cells did not show an increase in the cellular expression of the viral Spike (S) protein from 6 h P.I., thus indicating that the drug inhibits SARS-CoV-2 replication. Reasonably, the release of viral particles, which increased 10 h and 24 h following infection of Calu-3 cells, was strongly reduced when viral replication was previously inhibited by the addition of Tetrandrine (Fig. 2C).

Moreover, since we have previously demonstrated that Tetrandrine is able to modulate autophagy in a dose-dependent manner, we asked whether it is also the case in SARS-CoV-2 BA.5 infected cells. To test this hypothesis, we quantified autophagic vesicles by measuring the area of LC3B-positive puncta per cell by immunofluorescence following infection, as suggested by guidelines [27]. Our results demonstrated that cellular LC3 strongly increases in Tetrandrine treated cells following viral infection, 5 µM being more effective than 10 µM (Fig. 2D). This result suggests Tetrandrine was able to impact autophagy regulation in infected cells, even though SARS-CoV-2 infection did not seem to substantially impact autophagy in our cellular model when compared to untreated groups. Interestingly, it has been reported that SARS-CoV-2 viral particles localize on autophagic vesicles by analyzing the colocalization between viral Spike and LC3B puncta by confocal microscopy [7, 28, 29]. In this context, a pivotal role is probably mediated by virale Spike, indeed recent reports suggest that pseudoviruses, which contain ΔC1-19 Spike, enhances the initial recruitment of Syntaxin-6 (STX6) to early endosomes, promoting the trafficking of the virus toward the autophagic vesicles [30]. Furthermore, the association is probably mediated by the direct interaction between the viral M protein with LC3B [31]. Based on this evidence, we were interested in evaluating whether the antiviral effect of Tetrandrine is achieved through perturbing viral localization on autophagic vesicles. Therefore, we assessed whether viral particles are sequestered into autophagic vesicles, by measuring the co-localization of viral Spike (S) and LC3B signals, respectively and by using Mander’s coefficient. As reported in Fig. 2E, in our experimental conditions, viral particles localized on autophagic vesicles 3 h P.I., then this association is progressively reduced during infection in untreated cells, in line with the viruses released (Fig. 2C, blue line). The addition of Tetrandrine, which in turn increases LC3B puncta and blocks viral replication, did not show this trend. These results suggest that Tetrandrine could also impact viral particle release through autophagy. To test this hypothesis, we initially analyzed whether Tetrandrine modulates autophagy in a dose-dependent manner following infection, as previously shown in infected cells (Supplementary Fig. 1B). To this aim, we evaluated autophagic flux 24 h post SARS-CoV-2 infection in the presence or absence of Bafilomycin A1 and following treatment with Tetrandrine at both concentrations. As reported in Fig. 2F, the western blotting analysis of the autophagic markers LC3B and the relative densitometric analysis showed that autophagy was strongly activated by Tetrandrine 24, at both concentrations, 24 h after the infection with the BA.5 variant of SARS-CoV-2. Altogether, our results indicate that the addition of Tetrandrine 2 h before SARS-CoV-2 infection not only inhibits viral replication, but also activates autophagy maintaining SARS-CoV-2 viral particles on the autophagic vesicles for prolonged times.

### Autophagy and tetrandrine treatment may affect SARS-CoV-2 infection independently of each other

**Fig. 3:**
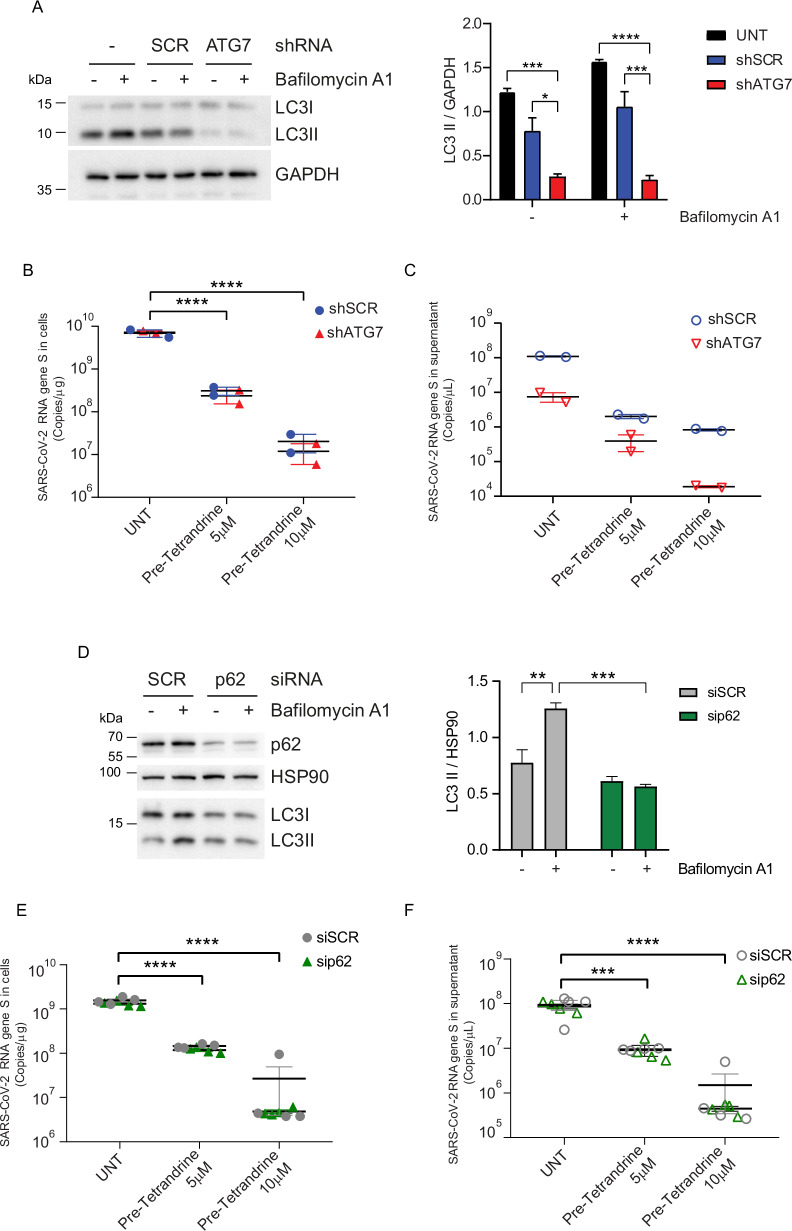
Analysis of BA.5 variant viral replication in Calu-3 cells with ATG7 silencing and treated with Tetrandrine. Calu-3 cells with stable ATG7 silencing were pre-treated with Tetrandrine at 5 and 10 µM and subsequently infected with SARS-CoV-2 in the presence or absence of Tetrandrine for 24 h. **A** Western blot and densitometric analysis of LC3-II/GAPDH levels in Calu-3 cells transduced with control (shSCR) or ATG7 shRNA, with or without Bafilomycin A1. Quantification of SARS-CoV-2 RNA in cell lysates **B** and supernatants **C**. **D** Western blot and quantification of LC3-II/HSP90 in cells transfected with control (siSCR) or p62 siRNA indicate reduced autophagic flux upon p62 silencing. SARS-CoV-2 RNA levels in cell lysates **E** and supernatants **F**. All results are expressed as mean ± SEM and analyzed by one-way ANOVA followed by Tukey’s post hoc test. **p < 0.004, ***p < 0.0002 and ****p < 0.0001 compared to the UNT group.

Prompted by our results, we hypothesized that the anti-viral effects of Tetrandrine could be mediated by the strong activation of autophagy observed following SARS-CoV-2 infection. ATG7, a key autophagy-related protein required for autophagosome formation, has also been described to regulate Tetrandrine- induced autophagy in human hepatocellular carcinoma context [32]. Therefore, to test whether the antiviral effect of Tetrandrine depends on its ability to promote autophagy, we silenced ATG7 expression in Calu-3 by shRNA approach. As expected, the ATG7 downregulation (Supplementary Fig. 3A) leads to impaired LC3B lipidation following Bafilomycin A1 treatment, thus confirming the autophagy-deficient condition (Fig. 3A). Then, to evaluate the effect of canonical autophagy in orchestrating the Tetrandrine- mediated antiviral response, ATG7-silenced Calu-3 cells were infected with the SARS-CoV-2 Omicron BA.5 variant (MOI 0.2) and treated with Tetrandrine at different concentration 24 h P.I. The amount of S viral gene expression was measured by qPCR both in cells and in the supernatant (Fig. 3B and C). As shown in Fig. 3B, the addition of Tetrandrine at 5 and 10 µM causes a substantial reduction of viral Spike expression in the cells, which is maintained following ATG7 downregulation, thus suggesting Tetrandrine inhibits viral replication in an autophagy-independent manner. By contrast, the analysis of the S gene in the supernatant (Fig. 3C) revealed that autophagy inhibition impacts viral particles released in Calu-3 cells, a mechanism that appeared uncoupled to the addition of Tetrandrine. Since it has been described that p62 is targeted by SARS-CoV-2 to usurp autophagy at early steps [6], we used a similar experimental approach to evaluate the role of another protein essential in autophagy. Silenced p62 Calu-3 cells were evaluated by western blotting to monitor p62 and LC3B levels by western blotting. As assumed, LC3B lipidation is partially inhibited by p62 downregulation (Fig. 3D). Moreover, the SARS-CoV-2 inhibitory effect of Tetrandrine does not require p62, since both cellular and released viral Spike gene can be detected by qPCR (Fig. 3E and F).

Altogether, our results suggest that the suppression of SARS-CoV-2 replication observed by exposing Calu-3 cells to Tetrandrine is only partially assessed by its ability to activate canonical autophagy, suggesting that other pathways are involved. However, autophagy per se seems to play a relevant role in regulating the SARS-CoV-2 cycle since its inhibition affects the release of viral particles.

### Proteomics analysis of SARS-CoV-2-infected Calu-3 cells

**Fig. 4:**
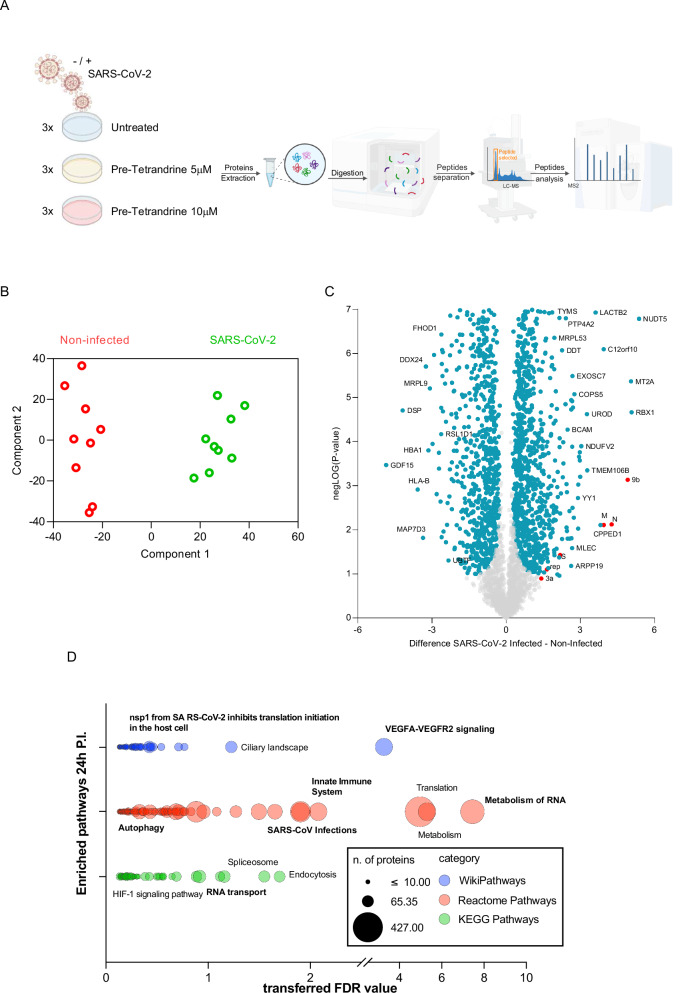
Proteomic analysis of Calu-3 cells infected with the SARS-CoV-2 BA.5 variant. Protein extracts derived from Calu-3 cells, either infected or uninfected for 24 h with the SARS-CoV-2 BA.5.1 variant and treated with Tetrandrine, were subjected to proteomic analysis. **A** Experimental workflow of the proteomic analysis; **B** Principal Component Analysis (PCA) showing the clustering of infected and uninfected cells; **C** Volcano plot displaying the 1,775 differentially abundant proteins (DAPs) between SARS-CoV-2-infected cells (right) and uninfected cells (left). Cellular proteins are shown in blue, while viral proteins are shown in red; **D** Pathway enrichment analysis performed using the STRING-App in Cytoscape, selecting WikiPathways, Reactome, and KEGG pathways.

Since Tetrandrine retained antiviral efficacy even in ATG7 and p62-silenced cells, we reasoned that additional, autophagy-independent mechanisms must contribute to its activity. To identify these alternative pathways, we performed quantitative proteomic profiling of Calu-3 cells infected with the SARS-CoV-2 Omicron BA.5 variant (MOI 0.2) and treated with Tetrandrine under the pre-treatment protocol, which had shown the highest antiviral efficacy in our earlier experiments. Protein extracts derived from three independent experiments were analyzed by mass spectrometry (MS)-based expression proteomics (Fig. 4A), and protein identification was performed using MaxQuant software, and the Principal Component Analysis of the entire dataset was performed using Perseus software (Fig. 4B and Supplementary Fig. 4A).

As shown in Fig. 4B, samples cluster into two main groups represented by infected and not infected cells, thus indicating the presence of SARS-CoV-2 was the main factor influencing protein abundant among the two different populations. Accordingly, the Volcano plot of differentially abundance proteins (DAPs) following SARS-CoV-2 infection (Fig. 4C) showed viral proteins enriched only in infected cells (red dots), and 1775 cellular proteins (blue dots) are differentially abundant following SARS-CoV-2 infection. As shown, by the Bubble plot reported in Fig. 4D, Reactome, KEGG, and Wiki pathways analyses indicated a strong modulation of mechanisms involved in SARS-CoV-2 infection, which included RNA metabolism, innate immune response, and autophagy. Therefore, our initial analysis of global proteomics-derived data underlines that the experimental settings used allow the clear discrimination of SARS-CoV-2 infected and non-infected cells, supporting the evaluation of cellular responses and pathways affected by the virus.

### Tetrandrine modulates the proteome of SARS-CoV-2 infected Calu-3 cells

In line with previous results, the unsupervised hierarchical cluster analysis confirmed that SARS-CoV-2 infection distinguishes the two main sample groups (Supplementary Fig. 5A). Interestingly, the analysis highlighted three subgroups of Tetrandrine treatments only in SARS-CoV-2 exposed cells, indicating that Tetrandrine did not significantly modulate protein expression in basal condition but mainly following infection.

**Fig. 5:**
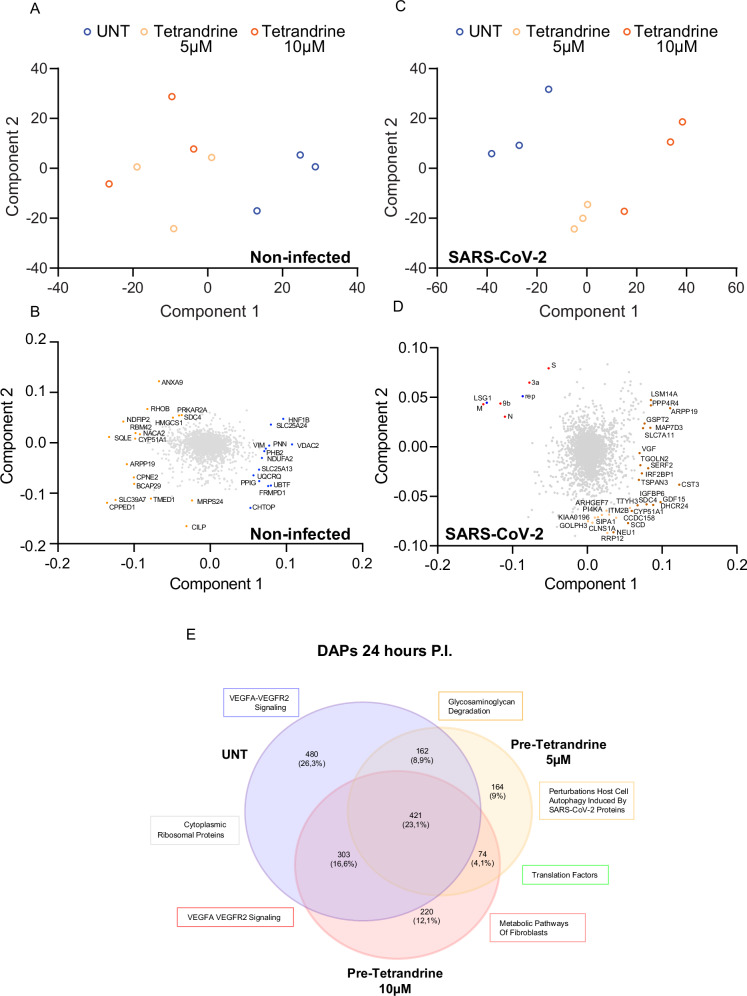
Proteomic analysis of SARS-CoV-2-infected Calu-3 cells based on Tetrandrine treatment. **A**–**D** Principal Component Analysis (PCA) plots showing global proteomic distribution across experimental groups under non-infected **A**, **B** and SARS-CoV-2-infected **C, D** conditions. Distinct clustering patterns reveal dose-dependent effects of Tetrandrine (5 µM and 10 µM) compared with untreated (UNT) controls. In non-infected cells **A**, treatment with Tetrandrine induces distinct proteomic signatures. In contrast, in SARS-CoV-2-infected cells **C**, the separation between clusters is more prominent, indicating a pronounced impact of Tetrandrine on virus–host protein networks. Panels **B** and **D** highlight the principal proteins contributing to the variance in each condition. **E**Venn diagram of differentially abundant proteins (DAPs) 24 h post infection identified in SARS-CoV-2-infected cells following Tetrandrine treatment.

Based on the prior premises, we deeply analyzed infected and non-infected groups separately based on Tetrandrine treatment. The principal component analysis reported in Fig. 5A and B confirmed that Tetrandrine treatment partially impacts the abundance of proteins in cells not exposed to viral infection; as shown, a slight separation of the untreated population from the Tetrandrine-treated cells could be observed independently from the used Tetrandrine concentration. The partial difference can be observed among untreated and Tetrandrine-treated cells, independently form the concentration, with most representative proteins highlighted in Fig. 5B. By contrast, following SARS-CoV-2 infection, the untreated population strongly differs from the Tetrandrine ones, with distinct separation between 5 µM and 10 µM concentrations (Fig. 5C), and as stressed by distinctive proteins reported in Fig. 5D. To further evaluate how Tetrandrine treatment modulates protein levels following SARS-CoV-2 infection, we extracted from the whole dataset proteins whose abundances were significantly modulated 24 h P.I. The evaluation was performed for untreated, 5 and 10 µM Tetrandrine exposed cells, separately. The Venn diagram in Fig. 5E showed that 1824 proteins were differentially abundant (DAPs) following infection with SARS-CoV-2 and compared to non-infected cells. Considering the whole number of proteins, 164 were exclusive to the 5 µM group samples, 220 to the 10 µM group samples, and 480 specifics to the untreated group. Interestingly, 74 DAPs were found to be shared among the Tetrandrine treated samples independently from concentration.

### Tetrandrine modulates autophagy, cholesterol and insulin-like growth factor metabolism in SARS-CoV-2-infected Calu-3 cells

**Fig. 6:**
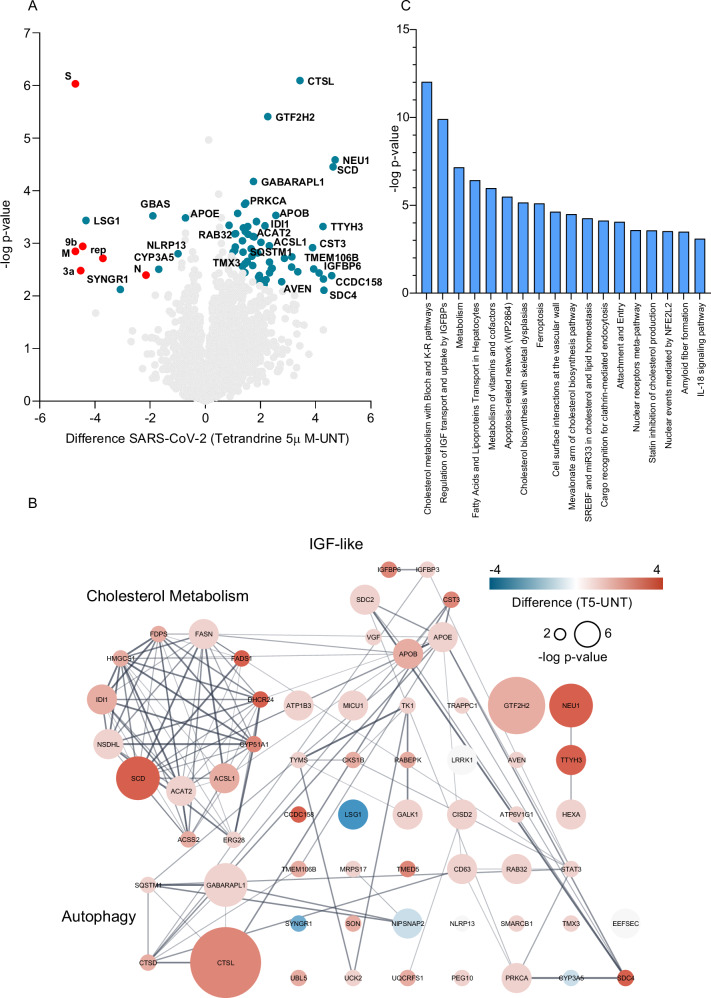
Signaling pathway analysis comparing SARS-CoV-2-infected Calu-3 cells untreated and pre-treated with Tetrandrine at 5 μM. **A** Volcano plot comparing untreated SARS-CoV-2-infected Calu-3 cells (left) and pre-treated cells with Tetrandrine at 5 µM (right). Cellular differentially abundant proteins (DAPs) are shown in blue, and viral proteins are shown in red; **B** Protein network analysis using STRING; **C** Pathway enrichment analysis performed using the STRING app in Cytoscape, with the WikiPathway, Reactome, and KEGG databases.

Since following SARS-CoV-2 infection 5 µM differed from the 10 µM Tetrandrine treatment, we compared single treatments to the untreated cells. Figure 6A shows the Volcano plot of DAPs from infected cells following 5 µM Tetrandrine treatment. As expected, SARS-CoV-2 proteins were expressed low following Tetrandrine treatment (red dots), supporting that the drug inhibits SARS-CoV-2 replication. Then, the network of all cellular DAPs was analyzed using the application of String-DB specifically developed for Cytoscape. The reported network illustrates protein abundance differences; the color indicates red for up- and blue for down-regulation in Tetrandrine, along with the relative statistical significance as represented by the intensity of the –log(p-value) (Fig. 6B). A Functional enrichment analysis was performed to identify pathways specifically modulated by 5 µM Tetrandrine treatment following SARS-CoV-2 infection. As shown in Fig. 6C, 15 pathways among KEGG, Reactome, and Wiki were modulated. In line with previous results, autophagy was altered by 5 µM Tetrandrine following infection. Unexpectedly, the metabolism of cholesterol and the insulin-like growth factor were also affected by Tetrandrine treatment.

**Fig. 7:**
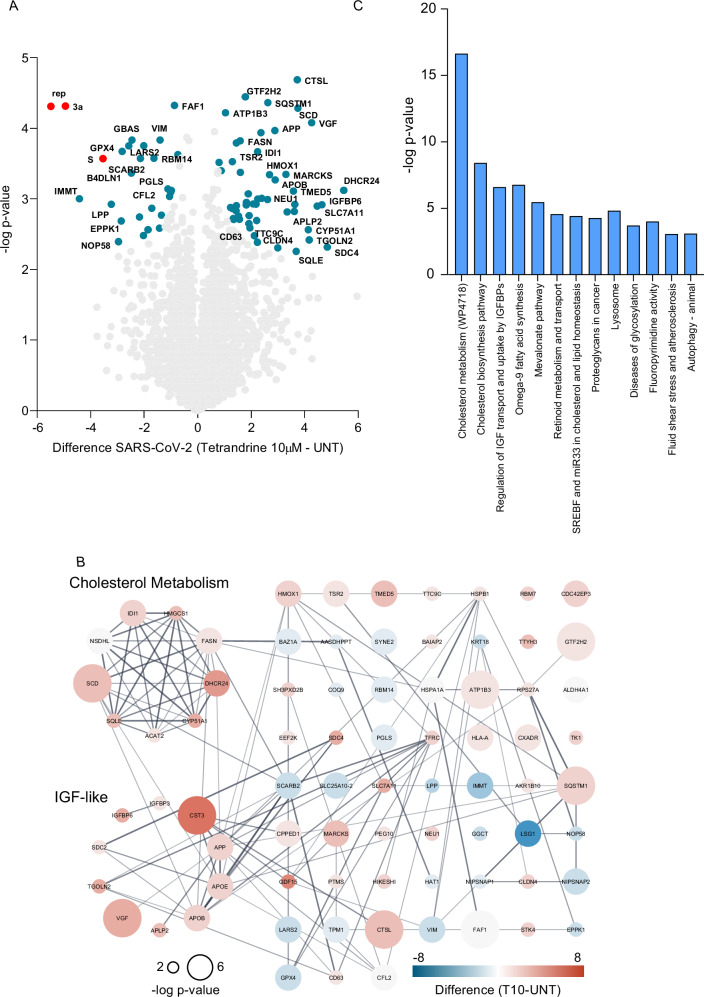
Signaling pathway analysis comparing SARS-CoV-2-infected Calu-3 cells untreated and pre-treated with Tetrandrine at 10 μM. **A** Volcano plot comparing untreated SARS-CoV-2-infected Calu-3 cells (left) and cells pre-treated with Tetrandrine at 10 μM (right). Cellular differentially abundant proteins (DAPs) are shown in blue, and viral proteins are shown in red; **B** Protein network analysis using STRING; **C** Pathway enrichment analysis performed using the STRING app in Cytoscape, with the WikiPathway, Reactome, and KEGG databases.

We have previously shown that both 5 µM and 10 µM Tetrandrine inhibited SARS-CoV-2 replication; therefore, we performed the same pathway analysis for 10 µM Tetrandrine treatment (Fig. 7). According to our previous analysis, also 10 µM Tetrandrine reduced the expression of SARS-CoV-2 proteins (Fig. 7A), further demonstrating Tetrandrine constrains viral replication. In addition, the network and the pathways analysis revealed cell adhesion and Notch3-mediated apoptosis were pathways specifically modulated by 10 µM Tetrandrine (Fig. 7B). However, both cholesterol metabolism and insulin-like growth factor were equally altered in 5 and 10 µM Tetrandrine exposition (Fig. 7C).

In summary, our results demonstrate that Tetrandrine acts as a multifaceted modulator of SARS-CoV-2 infection. The compound did not compromise Calu-3 cell viability at the tested concentrations, ensuring that its antiviral activity is not linked to cytotoxicity. Instead, it regulated autophagy in a dose-dependent manner, inducing flux at 5 µM and inhibiting it at 10 µM. Functionally, Tetrandrine significantly reduced viral replication, with pre-treatment proving more effective than post-treatment, thus suggesting an action at early stages of the infection cycle. Importantly, the drug did not block viral entry but instead impaired replication and release, partially by sustaining the association of viral particles with autophagic vesicles. Mechanistically, Tetrandrine inhibited viral replication even in ATG7- and p62-deficient cells, indicating that its effects are not fully dependent on autophagy. Proteomic analyses further revealed that Tetrandrine reshapes host pathways beyond autophagy, notably cholesterol metabolism and insulin-like growth factor signaling, both of which are essential for viral entry and replication.

Together, these findings indicate that Tetrandrine reduces SARS-CoV-2 infection through complementary mechanisms, acting both via autophagy modulation and through autophagy-independent pathways. These insights set the stage for a broader discussion on its therapeutic potential and mechanistic relevance.

## Discussion

One of the most impactful global health crises in modern history is represented by the COVID-19 pandemic, caused by the virus SARS-CoV-2, which has profoundly affected public health, economies, and daily life worldwide. One of the key aspects of SARS-CoV-2 pathogenesis resides in its capability to subvert host cellular mechanisms, including several processes of the immune and stress response (*e.g*. autophagy). Indeed, in steady-state conditions, autophagy operates at a basal level, continuously preventing the accumulation of toxic cellular components. However, it is rapidly upregulated in response to various stressors, such as nutrient deprivation, hypoxia, and infection [14]. During viral infections, autophagy is known to play a dual role. On the one hand, it serves as a defense mechanism by degrading viral components and presenting viral antigens to the immune system [33]; on the other hand, it acts as a hub for viral replication and survival [34]. In this regard, SARS-CoV-2 has been shown to manipulate autophagy in several ways. For instance, the virus can block the fusion of autophagosomes with lysosomes, thereby preventing the degradation of viral components and creating a favorable environment for viral replication [35, 36]. Moreover, SARS-CoV-2 proteins, such as NSP6, have been implicated in these processes, as they promote autophagosome formation while inhibiting their fusion with lysosomes [26]. By impairing the autophagic flux, the virus could evade host defenses and sustain its replication cycle [6]. Overall, the numerous interactions occurring between SARS-CoV-2 and autophagic proteins further highlight the virus’s ability to hijack cellular autophagy for its benefit, thus making it a potential target for COVID-19 therapy [14]. In this context, our study explored the molecular mechanisms by which Tetrandrine, a potent Ca^2+^ channel inhibitor [37], hampers SARS-CoV-2, with a particular focus on the role of autophagy in the viral life cycle.

Our results showed that Tetrandrine modulates autophagy in a dose-dependent manner in Calu-3 cells, a human lung adenocarcinoma epithelial cell line commonly used in SARS-CoV-2 research. At a lower concentration of 5 μM, Tetrandrine induces autophagy, as evidenced by the increased turnover of LC3-II, a marker of autophagosome formation. By contrast, at a higher concentration of 10 μM, Tetrandrine inhibits the autophagic flux, preventing the fusion of autophagosomes with lysosomes. Importantly, this interpretation was only possible when autophagic flux was assessed in the presence of the late-stage inhibitor Bafilomycin A1, since LC3-II levels alone can be misleading. This is in line with the established guidelines for monitoring autophagy [27], which recommends evaluating LC3 turnover in combination with lysosomal degradation inhibitors.

This dual effect is consistent with previous reports suggesting the capability of Tetrandrine to modulate autophagy in a dose-dependent manner, in which at lower concentrations the drug promotes autophagy by activating autophagy-related genes like ATG7 and through ROS generation and ERK activation in cancer cells model [35, 38]. At higher concentrations, it has been shown to inhibit autophagy by antagonizing TPC2, thus disrupting viral replication [36, 39]. In this context, we have shown that Tetrandrine significantly reduces SARS-CoV-2 infection in Calu-3 cells, and the antiviral effect is even more relevant when the cells have been pre-treated with Tetrandrine, thus indicating that the preventive treatment may enhance drug efficacy. Quantitative PCR analysis has revealed a substantial reduction in the levels of viral genes encoding the Spike (S) and Envelope (E) proteins in Tetrandrine-treated cells, with a more marked effect observed at the higher concentrations. Interestingly, the inhibitory effect of Tetrandrine on the SARS-CoV-2 shows similar trends for both cellular and supernatant-contained viral particles, thus suggesting that the viral replication could be more affected than its release. Recently, Liu et al. (2023) have demonstrated that Tetrandrine has more significant antiviral activity, particularly during the early-entry stage of SARS-CoV-2 wild-strain infection, preventing the virus from reaching the endolysosomes in Vero-E6 cells, even if the drug was pre-incubated before infection. The authors also pointed out that, as a Ca^2+^ channel blocker, Tetrandrine may hinder the maturation of trafficking-related processes through TPC2 and interfere with SARS-CoV-2 mobility within the cell, reducing viral entry. Our results suggest that the effects of Tetrandrine extend beyond just blocking TPC2 since we observed an increased number of viral particles in cell supernatant treated with Ned-19, a selective inhibitor of NAADP-induced Ca²⁺ release via TPC2 [40]. In addition, we have observed the anti-viral properties of Tetrandrine both pre- and post-virus incubation against the most circulating strain, *i.e*. omicron BA.5, which has already been described to infect host cells via endocytosis [41, 42]. Using this system, we have dissected how Tetrandrine impairs the viral life cycle by evaluating the SARS-CoV-2 amount inside the cells at different time points post-infection by confocal microscopy. At three hours post-infection, the S signal was similar between Tetrandrine-treated and untreated cells, thus suggesting viral particles could access cells independently from the treatment. On the other hand, our results show that Tetrandrine strongly reduced S signal six hours post-infection at both concentrations, thus supporting the fact that viral replication is mainly affected by the drug. In addition, we observed an increased number of autophagic vesicles following Tetrandrine treatment during infection at both concentrations, as revealed by LC3 immunofluorescence. Intriguingly, at ten hours post-infection, viral particle release increases with a corresponding reduction of Spike-LC3 co-localization in untreated cells, thus suggesting a possible link between autophagy modulation and virus shedding. In line, the addition of Tetrandrine to infected cells stably maintained the association of viral particles to the autophagic vesicles, further suggesting that autophagy could play a role in viral release rather than viral entry and replication. In this context, it has been shown that SARS-CoV-2 proteins (*e.g*. ORF7) activate LC3II leading to the accumulation of autophagosomes and then promoting the production of progeny virus [9]. Additionally, the disruption of autophagy and the over-accumulation of autophagosomes can impair the viral ability to hijack cellular resources necessary for its replication and lead to cell death [43, 44]. In line, we have observed that the inhibition of autophagy by ATG7 silencing per se reduces the released viral particles, which was independent of Tetrandrine treatment. Collectively, our results suggest that autophagy contributes to SARS-CoV-2 inhibition mediated by Tetrandrine to a lesser extent. Indeed, the inhibition of autophagy obtained by ATG7 downregulation does not impact on the antiviral effects of Tetrandrine. Together, our results suggest that Tetrandrine may interfere with additional pathways beyond autophagy, thus highlighting its multifaceted nature in antiviral activity.

Aimed at evaluating other pathways relevant to the anti-viral activity of Tetrandrine, we assessed the proteome of SARS-CoV-2 infected cells by mass spectrometry following the addition of Tetrandrine at different concentrations. Of note, despite Tetrandrine inhibition of SARS-CoV-2 replication being dose-independent, the two concentrations used in this study diversely impacted protein expression. This consideration prompted us to analyze the 5 µM concentration independently from 10 µM concerning untreated cells following infection. It is important to note that our data confirmed that Tetrandrine treatment strongly reduces the expression of viral proteins in both concentrations. Moreover, the data analysis identified several DAPs in Tetrandrine-treated and SARS-CoV-2-infected, which specifically belong to the cholesterol metabolism and insulin-like growth factor signaling, independently from Tetrandrine concentrations. It has been described that SARS-CoV-2 affects the insulin/IGF signaling pathway in the host cell/tissue modulating the infection [45]. On the other hand, the cholesterol pathway has been involved in SARS-CoV-2 entry and replication [46, 47] into the host cells; thereby, the capability of Tetrandrine to limit SARS-CoV-2 infection can reside in its ability to modulate these pathways when added before infection.

Given the well-established roles of cholesterol metabolism in viral entry and replication and the involvement of insulin/IGF signaling in the host response to infection, these pathways may represent critical nodes through which Tetrandrine exerts its antiviral effects [45, 47]. Importantly, cholesterol involvement in viral infection is not restricted to coronaviruses but it is a conserved host dependency exploited by diverse viruses. In HIV-1, virion-associated cholesterol is essential for maintaining viral particle integrity and infectivity, and cholesterol-rich lipid rafts facilitate viral entry, assembly, and budding [48, 49]. Although HSV has a distinct DNA replication cycle, it similarly depends on cholesterol-rich microdomains to stabilize viral envelopes and promote fusion with host membranes, underscoring cholesterol as a common requirement for enveloped viruses [50, 51]. In coronaviruses, cholesterol has been extensively described as critical for clathrin-mediated endocytosis and the integrity of endosomal and lysosomal membranes, processes necessary for genome release into the cytoplasm [52, 53]. Cholesterol also supports the formation of double-membrane vesicles that act as replication organelles in SARS-CoV-2 infection [54], as well as maintaining the integrity of the endosomal membrane, which is crucial for the fusion and release of viral contents into the cytoplasm [55]. Thus, by modulating cholesterol biosynthesis and transport, Tetrandrine may disrupt the availability of functional lipid rafts and endomembrane platforms required for viral entry, replication, and egress, offering a broad-spectrum antiviral mechanism conserved across unrelated viral families. In this regard, it has recently been reported that Tetrandrine inhibits LIMP-2-mediated cholesterol release from lysosomes [56], thus affecting its cytosolic availability. Overall, since several viruses, including SARS-CoV-2, usurp host cholesterol to support their life-cycle [57–59], the significant alterations in cholesterol disposal may exert antiviral effects by modifying intracellular viral trafficking, replication and assembly.

Among cholesterol metabolism, we found that Tetrandrine upregulates Cystatin C (CST3) and SDC2 during infection. Interestingly, these two proteins have been previously described in HIV and HSV replication in the host cells, suggesting that they are possibly conserved cellular targets in many viral infections [60, 61]. Moreover, the cholesterol synthetase DHCR24, which is induced by insulin, has been identified as downregulated in SARS-CoV-2-infected Calu-3 cells [62]. Our proteomic screening identified that DHCR24 protein levels increased upon Tetrandrine treatment, suggesting another possible mechanism for its antiviral properties. Similarly to cholesterol, we identified insulin-like growth factor signaling modulated by Tetrandrine during SARS-CoV-2 infection. In this regard, it has been recently suggested that insulin could potentially facilitate the entry and replication of SARS-CoV-2 in patients with diabetes [63]. At the same time, anti-diabetic drugs were shown to benefit diabetic patients with COVID-19 by suppressing mTOR signaling promoting autophagy and exerting anti-inflammatory effects [64]. These findings underscore the potential of Tetrandrine to target multiple pathways involved in SARS-CoV-2 infection, making it a versatile and promising therapeutic candidate.

**Fig. 8:**
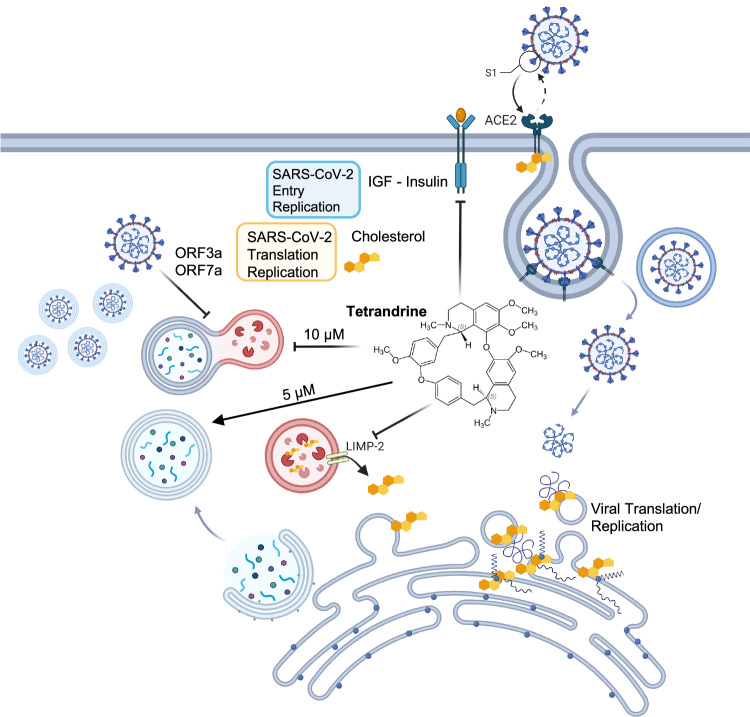
Proposed mechanisms of Tetrandrine action on the autophagic system, cholesterol metabolism, and IGF–Insulin signaling during SARS-CoV-2 infection. This schematic representation summarizes the potential cellular targets and dose-dependent effects of Tetrandrine during the SARS-CoV-2 life-cycle in host cells. Tetrandrine appears to interfere with the IGF–Insulin signaling pathway, which has been reported to inhibit mTOR and, consequently, promote autophagy induction via the canonical autophagic pathway. In parallel, the compound influences cellular cholesterol homeostasis by blocking lysosomal cholesterol release via LIMP-2. Since SARS-CoV-2 requires cytosolic cholesterol for replication and assembly, this effect may explain the antiviral properties of Tetrandrine. In addition, we reported that at a lower concentration (5 µM), Tetrandrine primarily induces autophagy, as shown by increased formation of autophagic vacuoles, suggesting an enhancement of autophagic flux which could aid in degrading viral components. Conversely, at a higher concentration (10 µM), Tetrandrine seems to impair the late stages of autophagy, likely by disrupting autophagosome-lysosome fusion or lysosomal function, resulting in the accumulation of autophagosomes. These findings suggest a concentration-dependent mechanism in which Tetrandrine differentially modulates autophagy during SARS-CoV-2 infection. Moreover, the alteration of the IGF–like axis and cholesterol metabolism induced by Tetrandrine may collectively contribute to the inhibition of SARS-CoV-2 translation and replication, emphasizing its multifaceted antiviral potential. (Prepared by the author with Biorender.com, 2025).

In summary, our study provides compelling evidence that Tetrandrine has the potential to inhibit SARS-CoV-2 infection through a combination of mechanisms, including the modulation of autophagy and interference with other cellular pathways essential for viral replication (Fig. 8). The observed dose-dependent effects of Tetrandrine on autophagy and its ability to impact viral replication, even in the absence of autophagy, highlight the complexity of its antiviral action. Given the multifaceted roles of autophagy in viral infections and the diverse pathways influenced by Tetrandrine, our study contributes to the broader effort of identifying effective treatments for SARS-CoV-2, with Tetrandrine emerging as a promising candidate for therapeutic intervention against this ongoing global health crisis.

Overall, while preclinical data are encouraging [65], comprehensive clinical evaluation is crucial to validate the efficacy and safety of Tetrandrine in COVID-19 patients. The complexity of SARS-CoV-2 pathology and the multifaceted roles of autophagy in viral infections underscore the need for a nuanced understanding of how Tetrandrine modulates these processes. By unraveling the complex interactions between Tetrandrine, autophagy, and viral replication, our research contributes to the broader scientific effort to develop effective treatments for SARS-CoV-2, ultimately aiming to mitigate viral activity and long-term symptoms. Future studies should focus on detailed clinical trials to explore the therapeutic potential of Tetrandrine in patients with COVID-19 and long-COVID. In addition, we found that Tetrandrine is more efficient in blocking SARS-CoV-2 replication when administered before infection, thus opening the possibility of evaluating its efficacy in preventing SARS-CoV-2 infection. This will provide crucial insights into its role as a potential antiviral agent and its application in the broader context of viral therapeutics.

## Materials and methods

### Drugs

Tetrandrine was purchased from Cayman Chemical, Ann Arbor, MI, USA; Bafilomycin A1 from St. Louis, Missouri, EUA; NAADP-AM was kindly provided by Grant Churchill (Department of Pharmacology, University of Oxford, UK) and Trans-Ned-19 (Tocris Bioscience, Bristol, UK) and Tetrandrine (Cayman Chemical, Ann Arbor, MI, USA).

### Cell culture

The human lung cancer cell line, Calu-3, was used as a cellular model for the present study. The cells were obtained from ATCC, tested mycoplasma-free using the Mycoplasma PCR Detection Kit (Abm, G238), and have not been recently authenticated. Calu-3 were cultured in *Eagle’s Minimum Essential Medium* (MEM; Sigma-Aldrich 51412 C) integrated with 10% of Fetal Bovine Serum (FBS), L-glutamine 2 µM (Corning 25-005-CI) and 1% of penicillin/streptomycin (Corning 30-001-CI). Cells were maintained in culture in sterile polystyrene plates, at 37°C and 5% of CO_2_ in thermostatic incubators. At 80–90% confluence the cells were diluted following this procedure: washed twice with PBS (Dulbecco’s Phosphate Buffered Saline; Euroclone ECB 4004 L), detached from the plate using trypsin/EDTA (Sigma-Aldrich T3924) and, finally dilute 1:4 to new plates (Corning).

### SARS-CoV-2 infection

SARS-CoV-2, human, Italy, clade GRA, lineage BA.5.1, Omicron (BA.5-like), strain hCoV-19/Italy/LAZ-INMI-3329/2022, former VOC Omicron GRA (B.1.1.529 + BA.*) first detected in Botswana/Hong Kong/South Africa was used to infect Calu-3 cells at a multiplicity of infection (M.O.I.) of 0.2. Viral inoculum or medium only (Not Infected) was added to cells and incubated for 1 h and 30′ at 37°C, 5% CO_2_. The viral inoculum was then removed, and cells were washed twice with PBS. Next, complete MEM was added, and cells were cultured at 37°C, 5% CO_2_. Immediately after inoculum removal (T0) or 24 h post-infection, supernatants and cells were collected for the subsequent analysis.

### Real-time RT-PCR and viral quantification

Based on the manufacturer’s guidelines, total RNA was isolated from Calu-3 cells with the Direct-zol RNA MicroPrep KIT (Zymo Research Corp). To perform RT-PCR analyses, 1 μg of total RNA was reverse transcribed using AMV-reverse transcriptase (Promega Corporation) to obtain single-stranded cDNA. To analyze the supernatant, RNA was extracted from 140 μL of Calu-3 culture medium using the Qiamp viral RNA kit (Qiagen) and then eluted in 50 μL of elution buffer. To determine viral abundance, the RealStar® SARS-CoV-2 RT-PCR Kit RUO (Altona Diagnostics) was used to amplify the E− and S- SARS-CoV-2 genes by Real-time qPCR using 10 μL of RNA extracted from supernatant, or 40 ng of cellular isolated RNA.

### ATG7 and p62 downregulation

For stable human ATG7 mRNA interference, a lentiviral ATG7 mRNA–targeting pLKO.1 plasmid was used (TRCN0000007584, Sigma-Aldrich) to produce lentiviral particles as follows: 293 T cells were transiently transfected with expression vectors using the calcium phosphate method with 10 mg of lentiviral vectors, 2.5 mg of pCMV-VSV-G, and 7.5 mg of psPAX2 plasmid for 48 h. Lentiviral particles in the supernatant were pelleted by ultracentrifugation at 19,800 RPM on an SW28 rotor for 2 h and resuspended with 500 mL PBS every 20 mL of collected medium. 300’000 Calu-3 cells were infected twice by adding 20 mL of viral suspension to complete MEM supplemented with polybrene (4 mg/mL) for 6 and 18 h, respectively. Following infection, cells were maintained in culture, and ATG7 expression levels were tested by Western blotting. p62/SQSTM1 was silenced by RNA interference using the RNA oligonucleotide duplex from Life Technologies (HSS113116), as per the manufacturer’s instructions.

### Western blots

Cells were lysed in CelLytic reagent (Sigma-Aldrich) completed with specific inhibitors: proteases inhibitor cocktail (PIC, Sigma Aldrich), phosphatases like sodium orthovanadate (Na3VO4) 0,5 µM, sodium molybdate (Na2MoO4) 5 µM, sodium fluoride (NaF) 5 µM, phenylmethanesulfonyl fluoride (PMSF) 0,5 µM and 1,10-phenanthroline (OPT) 2 µM and 2-chloroacetamide (ClCH2CONH2) 50 µM. Protein concentration was quantified using the BCA method (Thermo Fisher), and an equal amount of total protein per sample was mixed with a reducing agent (NuPAGE™ Cat. N. NP0004, Thermo Fisher) and Leamli Sample Buffer (Bio-Rad Cat. N. 1610747) and then loaded in homemade polyacrylamide gels at different concentrations for the SDS-PAGE. Proteins were transferred onto a PVDF membrane (Polyvinylidene Fluoride, Millipore) previously activated with 100% methanol, in semi-dry conditions using Transfer Blot Turbo (Bio-Rad) with a buffer consisting of 0.025 M Tris-base, 0.19 M glycine (TrisGly Bio-Rad, Cat N. 1610734) and 20% methanol. The filter was washed three times with PBS-Tween (0,1%) (T-PBS) and incubated with 5% milk solution in T-PBS for one hour to saturate non-specific sites. Incubation with the primary antibody specific to the protein of interest was performed overnight, at 4°C and stirring. The primary antibodies were diluted in 5% milk in T-PBS as indicated. After incubation, the primary antibody was removed from the filter and washed three times with T-PBS. Subsequently, a second one-hour incubation was performed using a specific peroxidase-conjugated secondary antibody diluted 1:5000 in 5% milk in T-PBS. The secondary antibody was removed, and the filter was washed three times with T-PBS. For protein detection, the PVDF membranes were incubated for three minutes with a commercial solution (ECL plus Millipore WBLUC0500 or WBLUR0500) containing the substrate for the chemiluminescence reaction catalyzed by peroxidase. Finally, the light signal was detected through a CCD camera of the ChemiDoc Touch Imaging System (Bio-Rad) associated with a digital image acquisition system. Image Lab 6.1 software from Bio-Rad was used to visualize and process images. Primary antibodies used were: Rabbit anti-LC3 (1:1000, Cell Signaling Technology 2775), Mouse anti-p62 (1:3000, MBL, M162-3), Mouse anti-GADPH (1:500000, Calbiochem CB1001), Mouse anti-HSP90 (1:1000, Santa Cruz Biotechnology, sc-13,119), Goat anti-ATG7 (1:500 Santa Cruz Biotechnology, sc-8668). The secondary antibodies used (Jackson lab) were anti-rabbit (JI 711–036-152), anti-mouse (JI 715–036-150), and anti-goat (JI 705–036-147), all diluted 1:5000. Full-length Western blots are included in the supplementary material.

### Immunofluorescence

For immunofluorescence analysis, Calu-3 cells were grown on glass coverslips, treated with Tetrandrine and infected as described above, and then fixed with 4% paraformaldehyde. After fixing, cells were washed twice with PBS and permeabilized by incubation with 0.5% Triton for 10 min at room temperature. Following a double washing with PBS, the non-specific background was reduced by blocking cells with 10% donkey serum in PBS for 30 min. Then, cells were incubated with primary antibodies diluted in 1% donkey serum for 1 h at room temperature, re-washed, and incubated with secondary antibodies for 30 min in the dark. Finally, ProLong™ Gold Antifade Mountant (Thermo Fisher, cat. N. P36931) was added to assemble the glass coverslip on a glass slide, which was subsequently sealed. The images were acquired using LSM 900, Airyscan SR Zeiss confocal microscope. At least 30 cells per sample have been acquired and then analyzed using ImageJ Fiji software. In detail, we measured the area of viral Spike or cellular LC3 per cell by using the ‘Analyze Particles’ tool of Image J. To evaluate the Lc3-Spike colocalization, the Jacop ImageJ plug-in has been used to assess the Mander’s coefficient of Spike signal on LC3 one. Primary antibodies: Rabbit Anti-LC3 (1:200, Sigma-Aldrich, L7543), Mouse Anti- SARS-CoV-2 spike (1:200), GeneTex (Irvine, CA, USA). Secondary antibodies: anti-rabbit Alexa Fluor 488-conjugated (Thermo Fischer, A21206), anti-mouse Cy3-conjugated (Jackson ImunoResearch, 715–166-150).

### Sample preparation for mass spectrometry

For whole proteome analyses of Calu-3 cells, with and without infection of SARS-CoV-2 and with the different treatments, 10 mg of protein extract per sample were incubated with 1 µM DTT, heated for 10 min at 75°C followed by 5.5 µM IAA incubated for 10 min in dark at RT. Trypsin was added with a 1:100 ratio (Trypsin: protein, w/w), and samples were left for digestion overnight at 37°C. TFA was added drop-wise to completely precipitate sodium deoxycholate and centrifuged at 14000 rpm, 23°C. Supernatants containing the peptides were STAGE tip-purified and dissolved in buffer A (0.1% formic acid in MS grade water) for LC-MS/MS measurements.

### Mass spectrometry-based proteomic analyses

HF-X mass spectrometer in line with an EasyLC 1000 nanoflow-HPLC (Thermo Fisher Scientific) has been used for LC-MS/MS. Purified peptides were separated using a 60 min ramp on fused silica HPLC column tips (I.D. 75 μm, New Objective, self-packed with ReproSil-Pur 120 C18-AQ, 1.9 μm [Dr. Maisch] to a length of 20 cm) of a gradient composed by water plus 0.1% formic acid (buffer A) and increasing concentrations of 80% acetonitrile in water plus 0.1% formic acid (buffer B). The mass spectrometer was operated in data-independent mode. Each survey scan was performed with mass range m/z = 350–1200 and resolution: 120’000, then 28 DIA scans were performed (isolation width of 31.4 m/z), thus covering a total range of precursors from 350–1200 m/z (AGC target value: 3 ×10^6^, resolution: 30’000, and 27% of normalized collision energy) [63]. Protein identification and quantification were performed with Spectronaut software (v15.7, Biognosys) without imputation in direct DIA mode and using full-length Uniprot human (UniProt, 2022), SARS-CoV-2 (Uniprot, Severe acute respiratory syndrome coronavirus 2 (2019-nCoV)), and common contaminants databases. Perseus software (version 1.6.15.0) [64] has been used to analyze DIA results. Before performing statistical analyses, an obvious batch effect of the data of replicate 3 was removed by using the “remove batch effect” function of the Perseus software with the Limma option as method. Obtained protein quantity values were log2 converted and grouped to compare infected to non-infected cells filtered based on proteins identified in at least one group with a coverage of 90%. Missing values were imputed from normal distribution based on the total matrix (width 0.3 and downshift 1.3). The whole matrix of identifications was used to calculate the Principal Component Analysis (2 components FDR < 0.05). To generate the volcano plots, we set 250 randomizations based on two sides t-test (FDR < 0.05 and S0 = 0.1). Differentially abundant proteins between non-infected and SARS-CoV-2 infected cells were compared based on Tetrandrine treatment, and a quantitative VENN diagram was generated using the open access online software, Venny 2.1 (https://bioinfogp.cnb.csic.es/tools/venny/). Hierarchical clustering of identified proteins was performed after Z-score normalization, and multiple-sample test (ANOVA) and Post hoc Tukey’s HSD test of one-way ANOVA (FDR < 0.05) were calculated for proteins among groups. Euclidean distance was used for the comparison, and clusters were extracted using *n* = 4 (between columns) and *n* = 18 (between rows). Proteins identified from SARS-CoV2 infected cells showing significant fold changes following Tetrandrine treatments were considered to analyze cellular modulated pathways. The network analysis and the visualization of cellular proteins significantly modulated were performed using the application of String-DB specifically developed for Cytoscape (v3.8.2) (StringApp) [65]. In the reported networks, the difference in the color of the nodes represents a differential fold of changes in the expression of Tetrandrine-treated cells versus untreated ones. In addition, the diameter refers to the statistical significance of the identification represented as the negative log of the *p*-value. The pathways analysis was performed by selecting Rectome, KEGG, and Wiki-Pathways through the Functional Enrichment analysis in Cytoscape. The analysis was performed with DAPs following Tetrandrine treatment of SARS-CoV-2 infected cells (FDR < 0.05, and redundancy= 0.4); the length of the bars represents -log of *p*-values of reported histograms. When changing pathways were represented through BubblePlot, the diameter of the bubble indicates the number of identified proteins.

### Statistical analysis

Statistical analysis of the data obtained from the Western blot, MTS, Trypan Blue, and Real-Time qPCR was performed, verifying the normal distribution by the Shapiro-Wilk test, and testing the homogeneity of variances by Brown-Forsythe. When the assumption of equal variances was violated, Welch’s ANOVA was applied in place of the standard one-way ANOVA. Values are shown as the mean ± SEM of at least three independent experiments. Densitometric analysis of Western blotting was performed using Image Lab 5.2.1 (Bio-Rad), with the control ratio arbitrarily defined as 1.00. The confocal imaging acquisition was not affected by group, as the operator was blinded to the allocation. For immunofluorescence analysis, the normal distribution was verified by the Shapiro-Wilk test, and statistical analysis was performed using a 2-way ANOVA test with multiple comparison tests based on the variable number. All statistical analyses were performed using GraphPad Prism 9.0 software, considering significant *P* values < 0.05.

## Supplementary information

Supplementary Table

Supplementary Fig. 1

Supplementary Fig. 3

Supplementary Fig. 4

Supplementary Fig. 5

Original Western blot files

Supp. Info

## Data Availability

The datasets generated during and/or analyzed during the current study are available from the corresponding author on reasonable request.
