# Supplementary figures and images for: Tetrandrine-driven autophagy suppresses SARS-CoV-2 replication by modulating cholesterol and IGF signaling pathways
Source: Cell Death Discov. 2026 Jan 6;12:82. doi: 10.1038/s41420-025-02926-7 (PMC12877079; doi:10.1038/s41420-025-02926-7)

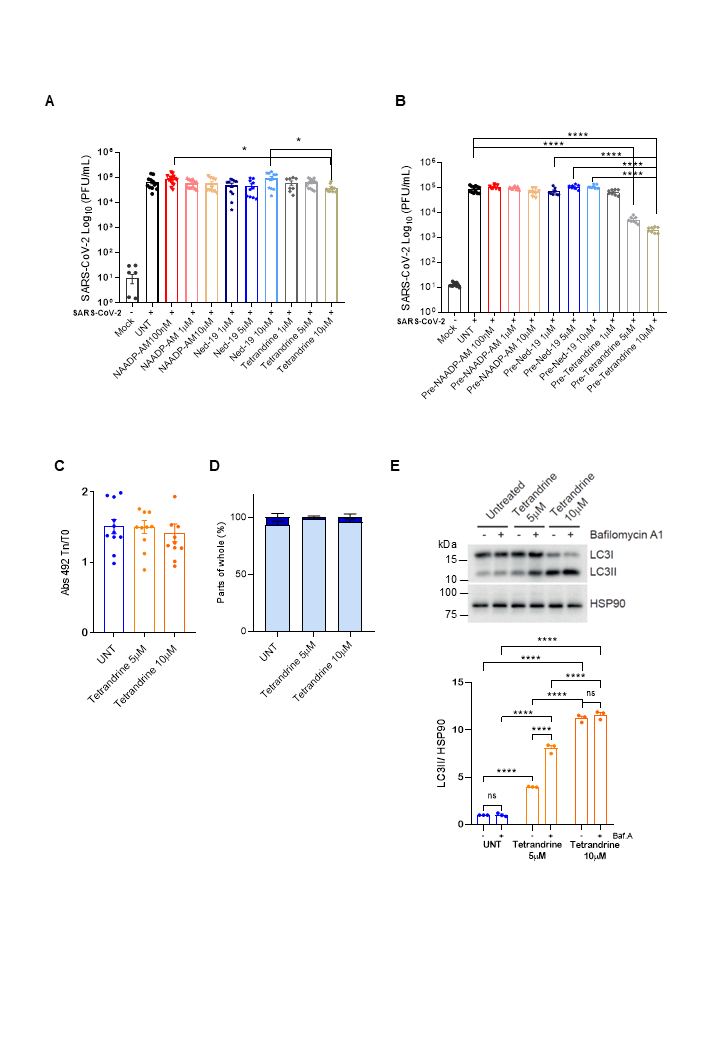

Supplement: Supplementary file 2 — Supplementary Fig. 1 [file 41420_2025_2926_MOESM2_ESM.tif]

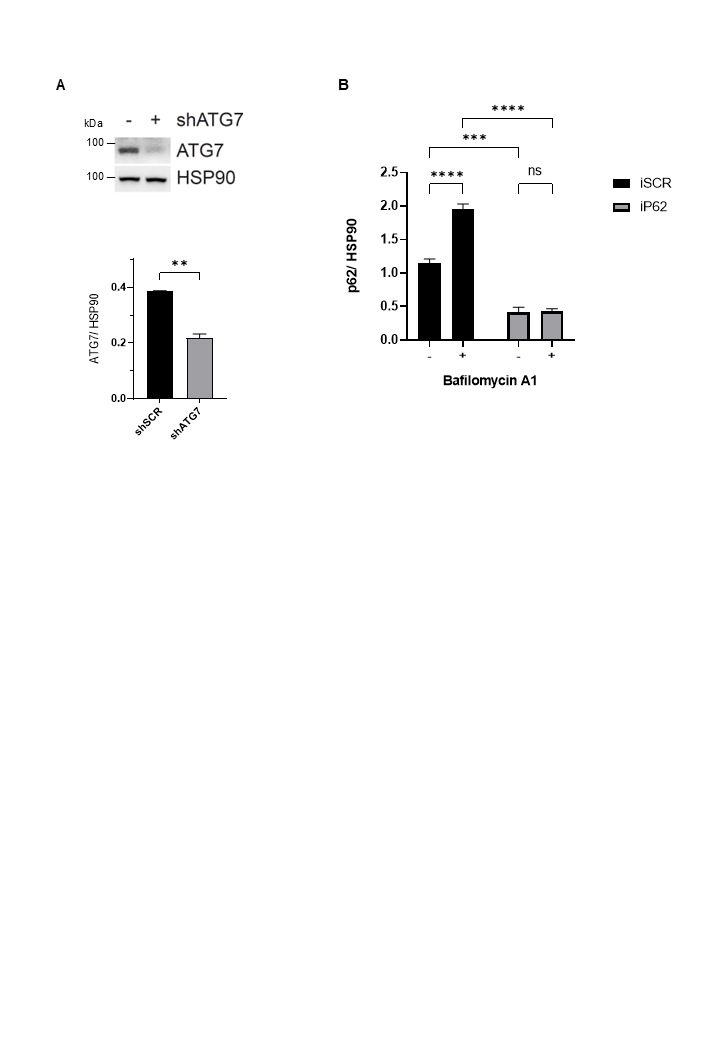

Supplement: Supplementary file 3 — Supplementary Fig. 3 [file 41420_2025_2926_MOESM3_ESM.tif]

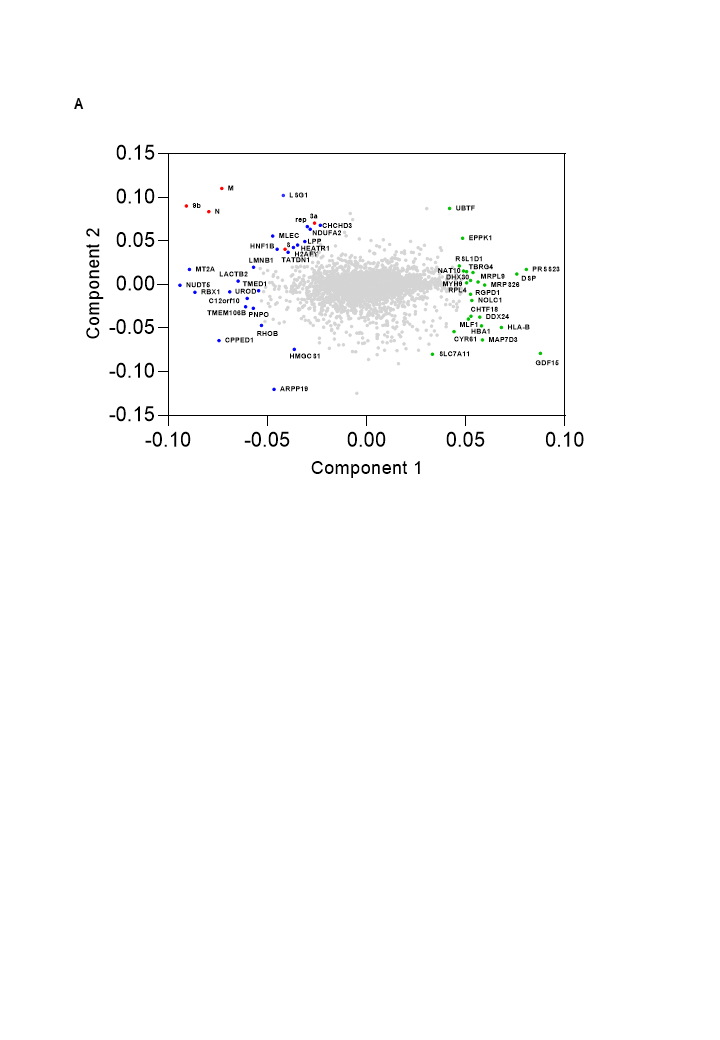

Supplement: Supplementary file 4 — Supplementary Fig. 4 [file 41420_2025_2926_MOESM4_ESM.tif]

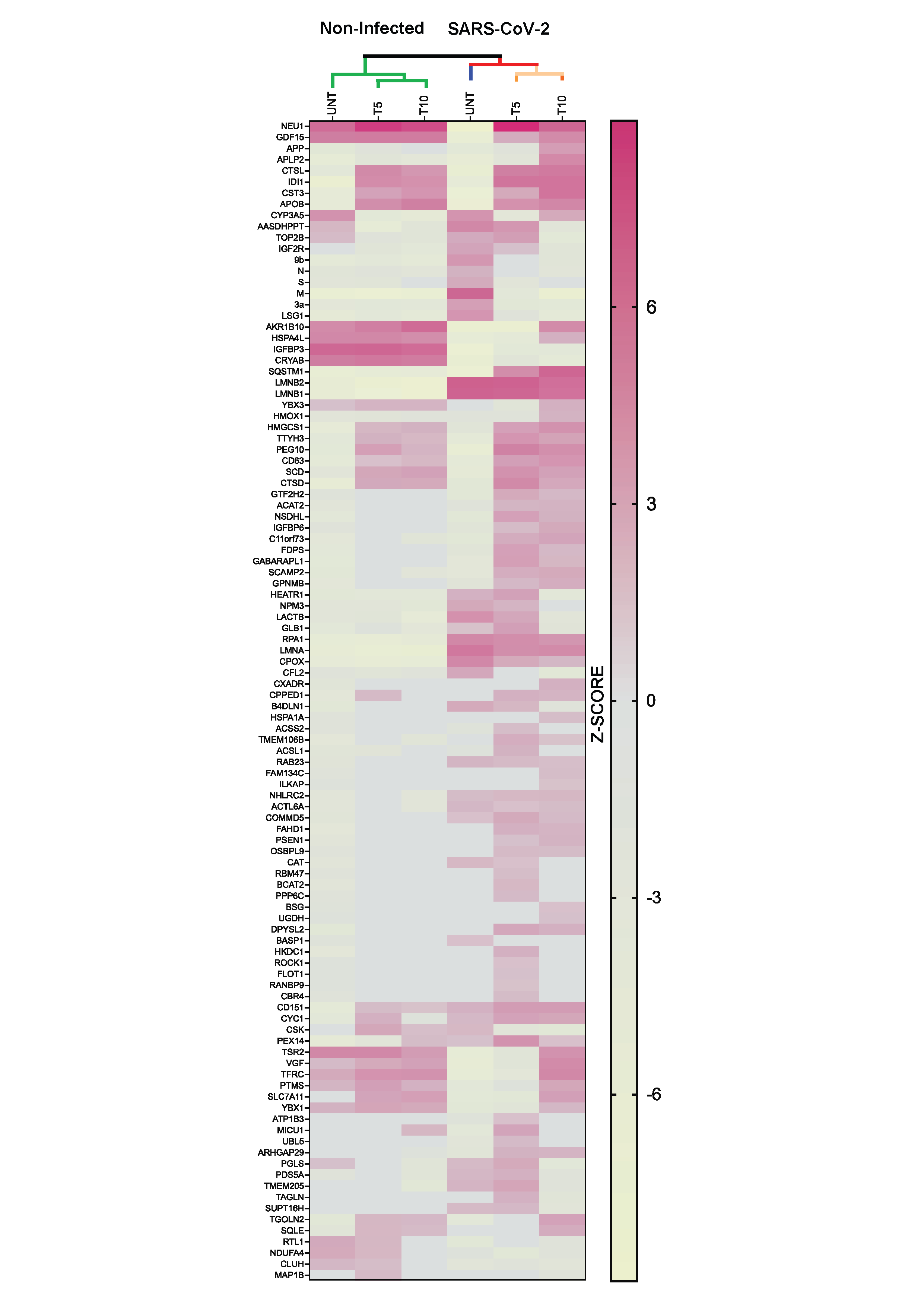

Supplement: Supplementary file 5 — Supplementary Fig. 5 [file 41420_2025_2926_MOESM5_ESM.tif]

**Figure 2**

**F**

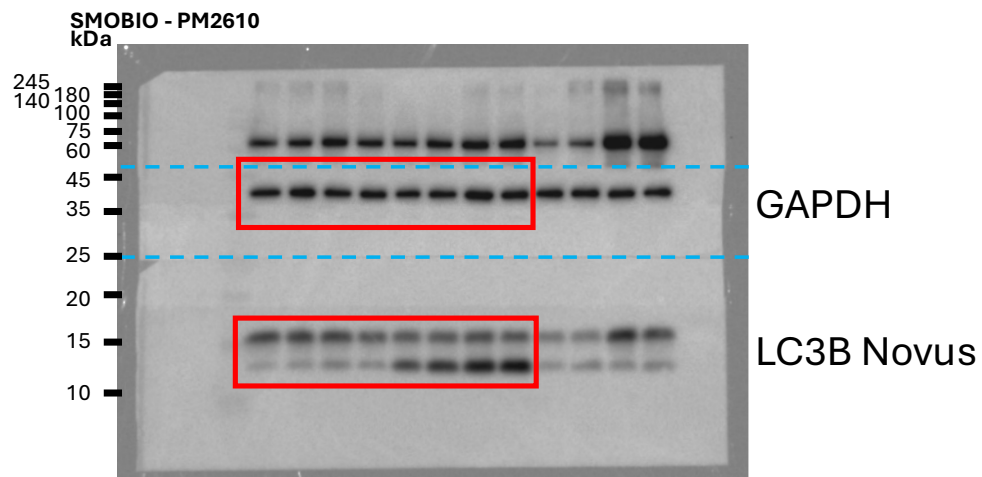

Figure 3

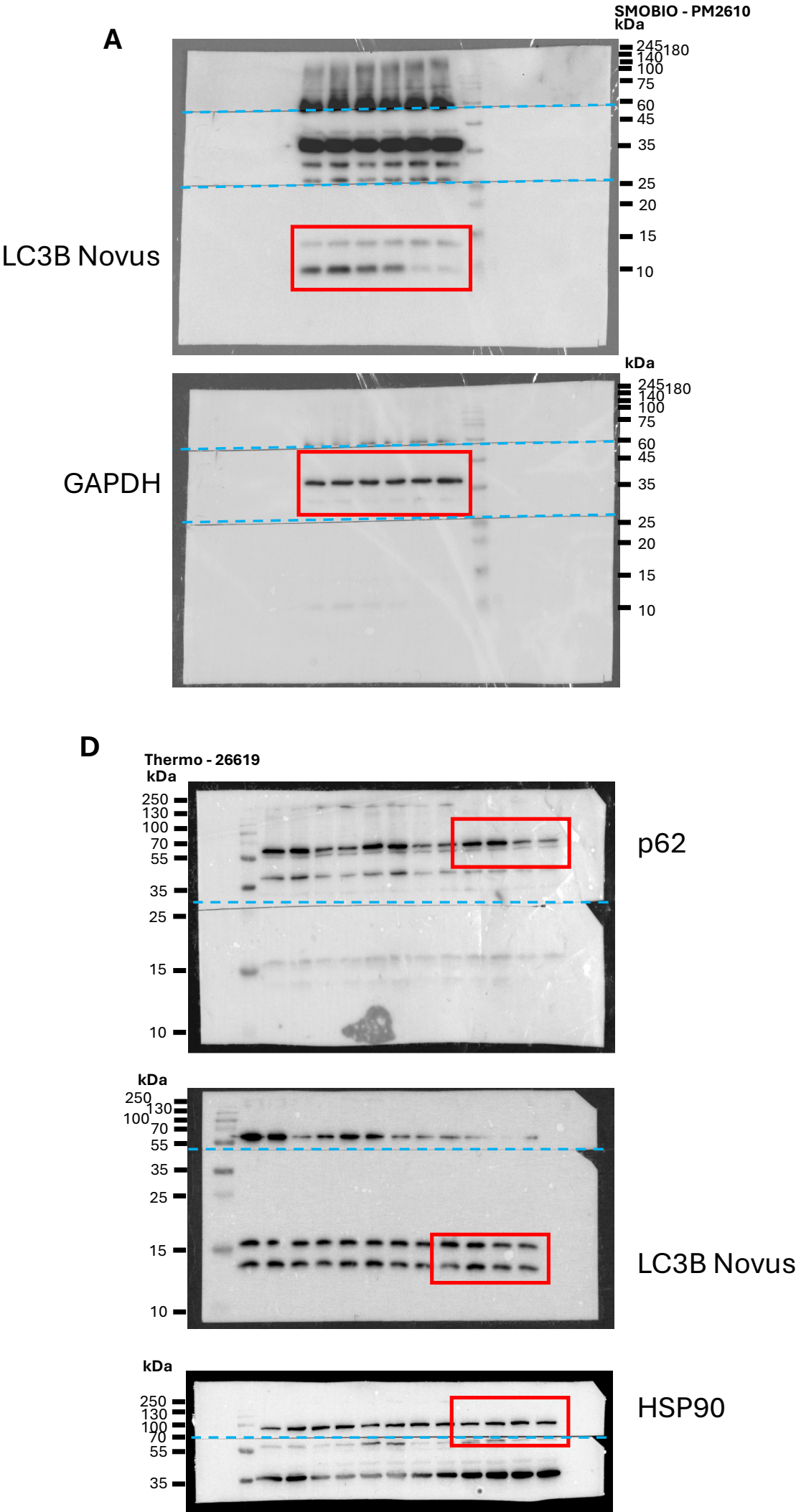

Supplementary Figure 1E

E

SMOBIO - PM2610

kDa

245

180

140

100

75

60

35

25

20

15

10

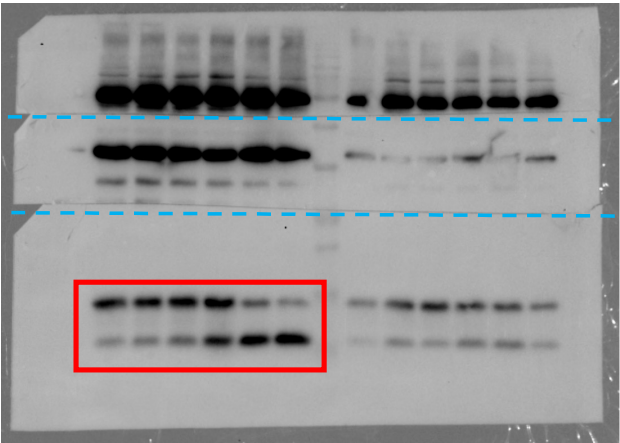

LC3B Novus

kDa

245

180

140

100

75

60

35

25

20

15

10

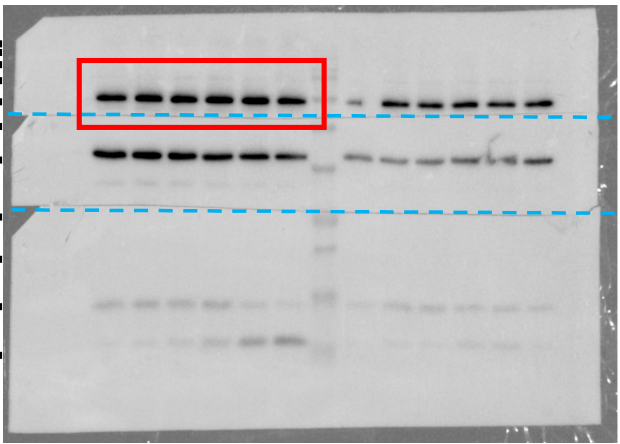

HSP90

Supplementary Figure 3A

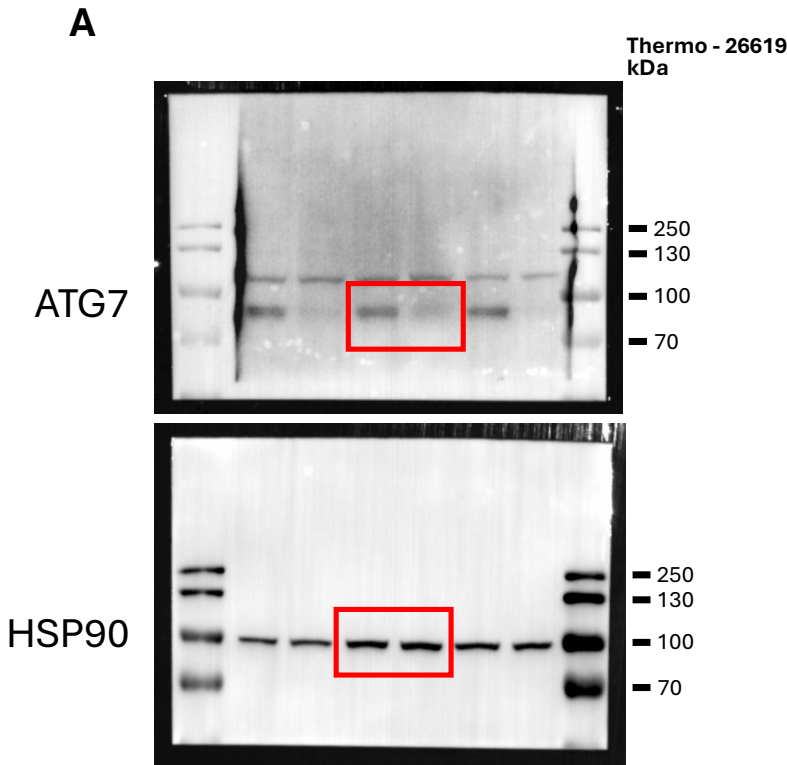

Supplement: Supplementary file 6 — Original Western blot files [file 41420_2025_2926_MOESM6_ESM.pdf]
