# Supplementary material for: Tetrandrine-driven autophagy suppresses SARS-CoV-2 replication by modulating cholesterol and IGF signaling pathways
Source: Cell Death Discov. 2026 Jan 6;12:82. doi: 10.1038/s41420-025-02926-7 (PMC12877079; doi:10.1038/s41420-025-02926-7)
Supplement: Supplementary file 7 — Supp. Info [file 41420_2025_2926_MOESM7_ESM.docx]

**Tetrandrine-Driven Autophagy Suppresses SARS-CoV-2 Replication by Modulating Cholesterol and IGF Signaling Pathways**

Lais de O. Marchioro (1, 2)^#^, Sofia De Stefanis (3)^#^, Beatriz G. Araújo (1), Davide Mariotti (4), Ingrid K. M. Watanabe (5), Michael Stumpe (6), Giulia Matusali (4), Fabrizio Maggi (2, 4), Soraya S. Smaili (1), Jörn Dengjel (6), Gustavo J. S. Pereira (1,7)^*^, Manuela Antonioli (2, 8)^*^

1. Department of Pharmacology, Escola Paulista de Medicina, Universidade Federal de São Paulo, São Paulo, SP, Brazil.
2. Department of Epidemiology, Preclinical Research and Advanced Diagnostics, National Institute for Infectious Diseases "Lazzaro Spallanzani"- IRCCS 00149 Rome, Italy.
3. PhD Program in Cellular and Molecular Biology, Department of Biology, University of Rome "Tor Vergata", 00133 Rome, Italy.
4. Laboratory of Virology and Laboratories of Biosafety, National Institute for Infectious Diseases "Lazzaro Spallanzani"-IRCCS, 00149 Rome, Italy.
5. Nephrology Division, Department of Medicine, Escola Paulista de Medicina, Universidade Federal de São Paulo, SP, Brazil.
6. Department of Biology, University of Fribourg, 1700 Fribourg, Switzerland.
7. Department of Cell and Developmental Biology, University College London, London, United Kingdom.
8. Department of Biology, University of Rome "Tor Vergata", 00133 Rome, Italy.

**Supplementary Information**

**Experimental Procedures**

**Drug.** NAADP-AM was kindly provided by Grant Churchill (Department of Pharmacology, University of Oxford, UK). The compounds Trans-Ned-19 (Tocris Bioscience, Bristol, UK) and Tetrandrine (Cayman Chemical, Ann Arbor, MI, USA) were commercially acquired. The MTT ([3-(4,5-dimethylthiazol-2-yl)-2,5-diphenyltetrazolium bromide]), used in the cell viability protocol, was purchased from Sigma-Aldrich (St. Louis, MO, USA).

**Cell culture.** Calu-3 were cultured in *Eagle’s Minimum Essential Medium* (MEM; Sigma-Aldrich 51412C) and Vero-E6 (*African green monkey kidney*; ATCC® CRL-1586™) cultivated in high-glucose Dulbecco's modified Eagle medium (DMEM, Sigma-Aldrich St. Louis, Missouri, USA), both mediums integrated with 10% of Fetal Bovine Serum (FBS), L-glutamine 2μM(Corning 25-005-CI) and 1% of penicillin/streptomycin (Corning 30-001-CI). Cells were maintained in culture in sterile polystyrene plates, at 37°C and 5% of CO_2_ in thermostatic incubators. At 80-90% confluence the cells were diluted following this procedure: washed twice with PBS (Dulbecco's Phosphate Buffered Saline; Euroclone ECB 4004L), detached from the plate using trypsin/EDTA (Sigma-Aldrich T3924) and, finally dilute 1:4 to new plates (Corning).

**Cell viability assay.** Calu-3 cells were plated in 96 wells plates (2500 cells/well). The following day, cells were treated with 5 µm, 10 µM Tetrandrine, or DMSO as negative control. 24h after the treatment medium was replaced with 100μL of fresh one, plus 20 μL of MTS solution (Promega CellTiter 96® AQueous One Solution Cell Proliferation Assay, G3580). Cells were incubated for 2h at 37°C and 5% CO_2_, and then the absorbance was measured by spectrophotometer at 492 nm of wavelength.

**Trypan Blue assay.** To estimate cell death, Calu-3 cells were plated in 12 wells plates (30000 cells/well) and left growing overnight. The following day, 5 m, or 10 mM Tetrandrine, or DMSO were added for 24 hours. Then, cells were washed with PBS, detached from the plate using trypsin, and stained with trypan blue solution diluted 1:2 with PBS. The amount of both stained and unstained cells was determined by using a Burker chamber. Live and death cells were expressed as the percentage of total cells counted per mL.

**SARS-CoV-2 infection.** SARS-CoV-2 B.1.1.7 strain (SARS-COV-2/HUMANA/BRA/HIAE-02/SP02/2020, GenBank MT126808) was kindly provided by Professor Edison Luiz Durigon from the University of São Paulo (USP), São Paulo-SP and used to infect Vero-E6 cells at a [multiplicity of infection](https://www.sciencedirect.com/topics/immunology-and-microbiology/multiplicity-of-infection) (M.O.I.) of 0.2. Viral [inoculum](https://www.sciencedirect.com/topics/biochemistry-genetics-and-molecular-biology/inoculation) or medium only (Not Infected) was added to cells and incubated for 1 h and 30′ at 37°C, 5% CO2. The viral inoculum was then removed, and cells were washed twice with PBS. Next, complete DMEM was added and cells were cultured at 37°C, 5% CO2. 24h post-infection, supernatants and cells were collected for the subsequent analysis.

Cell treatment. Vero-E6 cells were seeded in a 3,5 cm diameter plate (300’000 cells/well). The following day, for pre-treatment, Tetrandrine was added two hours before SARS-CoV-2 infection at the indicated concentrations, and, following the exposition to the virus, it was added again to the fresh medium. For post-treatment, Tetrandrine was added only to the fresh DMEM following SARS-CoV-2 exposition.

**Real-time PCR***.* RNA from 140 µL of cell culture supernatant was isolated using the QIAmp viral RNA kit (Qiagen, Germantown, MD, USA) following manufacturer’s instructions and eluted in 40 µL of elution buffer. To determine viral replication, Real time RT-PCR was performed using 10 µL of RNA according to the protocol described by Corman *et al.*(Corman et al., 2020) using the AgPath-ID One Step kit (Applied Biosystems, Waltham, Massachusetts, USA) in QauntStudio™ (Thermo Fisher Scientific, Waltham, MA, USA). The primers and probe sequences are the following: ESARBECO gene FW5’3’ ACAGGTACGTTAATAGTAATAGCGT, RV5’3’ ATATTGCAGCAGTACGCACACA and probe 6FAM-ACACTAGCCATCCTTACTGCGCTTCG-QSY.

**Western blotting.** Cells were treated according to each experimental protocol and lysed in CelLytic reagent (Sigma-Aldrich) in the presence of a cocktail of protease and phosphatase inhibitors (NaF (5 mM), NaMo (1 mM), NaOt (0.5 mM), PMSF (0.5 mM) and IP 1:100). Protein concentration was quantified using the BCA method (Thermo Fisher). To this aim, a standard curve of known BSA (bovine serum albumin; Sigma-Aldrich) amounts was used to determine the concentration of the extracted proteins. An equal amount of total protein per sample was mixed with a reducing agent (NuPAGE™ Cat. N. NP0004, Thermo Fisher) and Leamli Sample Buffer (Bio-Rad Cat. N. 1610747), and then loaded in homemade polyacrylamide gels at different concentrations for the SDS-PAGE sequentially transferred to PVDF membranes (Polyvinylidene Fluoride, Millipore) previously activated with 100% methanol. Protein transfer to the membrane was performed in semi-dry conditions at a constant voltage using Transfer Blot Turbo (Bio-Rad) with a buffer consisting of 0.025 M Tris-base, 0.19 M glycine (TrisGly Bio-Rad, Cat N. 1610734) and 20% methanol. The primary antibodies used were diluted in 5% milk in T-PBS as subsequently indicated and the secondary antibody diluted 1:5000 in 5% milk in T-PBS. For protein detection, the PVDF membranes were incubated for three minutes with a commercial solution (ECL plus Millipore WBLUC0500 or WBLUR0500) containing the substrate for the chemiluminescence reaction catalyzed by peroxidase. Finally, the light signal was detected through a CCD camera of the ChemiDoc Touch Imaging System (Bio-Rad) associated with a digital image acquisition system. Image Lab 6.1 software from Bio-Rad was used to visualize and process images.

Primary antibodies used were: Rabbit anti-LC3 (1:1000, Cell Signaling Technology 2775); Mouse anti-p62 (1:3000, MBL, M162-3), Mouse anti-HSP90 (1:1000, Santa Cruz Biotechnology, sc-13,119); Secondary antibodies used (Jackson lab) were anti-rabbit (JI 711–036-152), anti-mouse (JI 715–036-150) all diluted 1:5000.

**ATG7 and p62 downregulated Calu-3 cells.** For stable human ATG7 mRNA interference, a lentiviral ATG7 mRNA–targeting pLKO.1 plasmid was used (TRCN0000007584, Sigma-Aldrich) to produce lentiviral particles as follows: 293T cells were transiently transfected with expression vectors using the calcium phosphate method with 10 mg of lentiviral vectors, 2.5 mg of pCMV-VSV-G, and 7.5 mg of psPAX2 plasmid for 48 hours. Lentiviral particles in the supernatant were pelleted by ultracentrifugation at 19,800 RPM on an SW28 rotor for 2 hours and resuspended with 500 mL PBS every 20 mL of collected medium. 300’000 Calu-3 cells were infected twice by adding 20 mL of viral suspension to complete MEM supplemented with polybrene (4 mg/mL) for 6 and 18 hours, respectively. Following infection, cells were maintained in culture, and ATG7 expression levels were tested by Western blotting. For p62 downregulation, small RNA interference was performed using the HSS113116 RNA oligonucleotide duplex from Life Technologies.

**Supplementary Figures legends**

**Suppl. Figure 1. Evaluation of Infectivity in Vero-E6 Cells by SARS-CoV-2 (B.1.1.7) and after 24 Hours of Infection with or without 2-Hour Pre-Treatment and Cell Viability and Autophagic Modulation After 24 Hours of Treatment with Tetrandrine in Calu-3 Cells.** Expression of the ESARBECO gene after 24 hours of infection with SARS-CoV-2 (B.1.1.7) at an M.O.I. of 0.2, in the presence of treatments with NAADP-AM (100 nM), (1 µM) and (10 µM), Ned-19 (1 µM), (5 µM) and (10 µM), and Tetrandrine (1 µM), (5 µM) and (10 µM). Samples were normalized using the housekeeping gene Tubulin and the positive control (CTR+). **A:** post infection treatment protocol; **B:** pre infection treatment protocol. **C:** Assessment of cell viability using the MTT assay after 24 hours of treatment with Tetrandrine 5 µM and 10 µM or DMSO (UNT) in the Calu-3 cell line; **D:** Trypan blue exclusion test after treatments; **E:** Evaluation of autophagic flux in cells treated with Tetrandrine at 5 and 10 µM or DMSO (UNT) after 24 hours using Western blotting with anti-LC3 (14 kDa) and anti-HSP90 (90 kDa) antibodies. Bafilomycin A1 was added 2 hours before cell lysis. The optical density representing the relative expression of each protein was normalized using the internal control for the anti-HSP90 (90 kDa) antibody, as shown in the representations. All results are expressed as mean ± SEM, and data were analyzed by one-way ANOVA followed by Tukey’s post hoc test. *p < 0.03 compared to another treatment; ****p < 0.0001 compared to the UNT group.

**Suppl. Figure 3:** **Validation of ATG7 silencing in Calu-3 cells.** **A:** Representative Western blot showing ATG7 protein expression in Calu-3 cells transduced with shATG7 or control vector, using HSP90 as a loading control. Knockdown efficiency is evidenced by a marked reduction in ATG7 levels in shATG7 cells. Quantitative analysis confirming significant downregulation of ATG7 protein expression following shATG7 transduction compared to control. **B:** Quantitative analysis of p62 levels in Calu-3 cells silenced or not for p62 expression by siRNA, and treated or not with Bafilomicin A1 referred to the western blot panel in Figure 3D. All results are expressed as mean ± SEM, and data were analyzed by one-way ANOVA followed by Tukey’s post hoc test. *p < 0.03 compared to another treatment; ****p < 0.0001 compared to the UNT group.

**Suppl. Figure 4:** **Dot plots relative to the proteins loaded in the PCA in figure 4B.** The most relevant proteins in each area have been highlighted, left: enriched in SARS-CoV-2 infected cells and right: enriched in non-infected Calu-3 cells.

**Suppl. Figure 5: Heatmap and hierarchical clustering.** Heatmap reporting and unsupervised hierarchical cluster analysis of proteomics data; columns: experimental conditions, rows: identified proteins. Scale colors: Z-score normalization, followed by ANOVA and Tukey’s HSD post hoc tests (FDR < 0.05).
